# Glycosylation of Phenolic Compounds by the Site-Mutated β-Galactosidase from *Lactobacillus bulgaricus* L3

**DOI:** 10.1371/journal.pone.0121445

**Published:** 2015-03-24

**Authors:** Lili Lu, Lijuan Xu, Yuchuan Guo, Dayu Zhang, Tingting Qi, Lan Jin, Guofeng Gu, Li Xu, Min Xiao

**Affiliations:** State Key Lab of Microbial Technology and National Glycoengineering Research Center, Shandong University, Jinan 250100, PR China; National Cancer Institute at Frederick, UNITED STATES

## Abstract

β-Galactosidases can transfer the galactosyl from lactose or galactoside donors to various acceptors and thus are especially useful for the synthesis of important glycosides. However, these enzymes have limitations in the glycosylation of phenolic compounds that have many physiological functions. In this work, the β-galactosidase from *Lactobacillus bulgaricus* L3 was subjected to site-saturation mutagenesis at the W980 residue. The recombinant pET-21b plasmid carrying the enzyme gene was used as the template for mutation. The mutant plasmids were transformed into *Escherichia coli* cells for screening. One recombinant mutant, W980F, exhibited increased yield of glycoside when using hydroquinone as the screening acceptor. The enzyme was purified and the effects of the mutation on enzyme properties were determined in detail. It showed improved transglycosylation activity on novel phenolic acceptors besides hydroquinone. The yields of the glycosides produced from phenol, hydroquinone, and catechol were increased by 7.6% to 53.1%. Moreover, it generated 32.3% glycosides from the pyrogallol that could not be glycosylated by the wild-type enzyme. Chemical structures of these glycoside products were further determined by MS and NMR analysis. Thus, a series of novel phenolic galactosides were achieved by β-galactosidase for the first time. This was a breakthrough in the enzymatic galactosylation of the challenging phenolic compounds of great values.

## Introduction

β-Galactosidases (EC 3.2.1.23) occur in nature very frequently. They are widely distributed in plants and animals, as well as in a wide variety of microorganisms including yeasts, fungi, bacteria and archaea. These enzymes have attracted particular interest in the industrial applications owing to their hydrolase and transferase activities [1–3]. The hydrolytic activity has been applied in the food industry for decades for reducing the lactose content in milk to help prevent symptoms from lactose intolerance, while the transglycosylation activity has been used to synthesize prebiotic galacto-oligosaccharides from lactose [3–6]. Recently, interest in β-galactosidases has gained more momentum due to their production of promising galactose-containing chemicals, including diverse oligosaccharides, alkyl-glycoside, glycoconjugates and others that play important roles in the industries of food additives, cosmetics, and medicines [1].

β-Galactosidases produced the glycoside chemicals through galactosyl transfer from lactose or galactoside donors to various acceptors. The formation of glycosidic linkages mostly occurs between the galactose and the alcoholic hydroxyl groups of acceptors. Simple alkyl alcohols are good acceptors for the enzymes to produce alkyl-glycoside [7, 8]. Even complex compounds containing the alkyl-alcoholic side chains, such as the isotaxiresinol with anti-cancer activity, can be modified by the β-galactosidases from *E*. *coli* and *Kluyveromycs fragilis* [9]. The compounds bearing sugar hydroxyl groups are also common acceptors for the β-galactosidases. One example is that the β-galactosidases from *Bifidobacterium bifidum* and *Streptococcus* sp. 6646K are able to transfer glycosyl to *N*-acetylglucosamine (GlcNAc) or *N*-acetylgalactosamine (GalNAc), producing the β-1,3 or β-1,4 linkages of galactosyl GlcNAc/GalNAc that are related to ABO and tumor-associated carbohydrate antigens [10]. Another example is that the β-galactosidase from *Bacillus circulans* could glycosylate myricitrin, a complex flavonol rhamnoside with high anti-oxidative ability, resulting in 480 times more solubility in water [11]. Besides linear acceptors, cyclic tetrasaccharide also could be modified by the β-galactosidases from *Aspergillus oryzae* and *B*. *circulans*, forming 6' or 3' galactosyl cyclic tetrasaccharides that have the potential to be used as low-calorie sweeteners, drug-delivery carriers or affinity-chromatography materials [12]. Despite the flexibility of β-galactosidases toward acceptors, glycosylation of phenolic compounds were rarely reported.

The phenolic compounds are a class of chemicals with hydroxyl group bonded directly to the aromatic hydrocarbon group. Such chemicals have many physiological functions [13–17]. The catechins exhibit antioxidative and antibacterial activities, along with the capability of regulation of cholesterol levels in blood [14]. The caffeic acid has antioxidative, peroxy radical scavenging, and antimutagenic activities [15, 16]. Even simple phenolic compounds, such as phenol, hydroquinone, and catechol, play important roles as basilic intermediate materials that can be processed for valuable industrial chemicals or pharmaceuticals. Despite the importance of phenolic compounds, the use of them is usually limited because they are poorly soluble in water and also easily degraded by light irradiation in aqueous solution [14, 17].

Glycosylation of phenolic compounds is considered to be a very useful method to increase their solubility and stability in water [18, 19]. It can mask phenolic groups and protect them from oxidation [20]. Also, glycosylation can improve biological and pharmacological functions, including the decrease of toxicity and side effects, as well as the increase of bioavailability of drugs that need to pass through the blood-brain barrier [20]. Since galactose is an important constituent of the carbohydrate chains of glycoconjugates involved in a variety of biological recognition events [10], the synthesis of galactosyl phenolic derivatives would be of scientific and/or practical interest.

To date, only one phenolic compound, hydroquinone, has been reported to be galacosylated by the β-galactosidase from *Kluyveromyces lactis*, producing galactoside with 1.19 times higher antioxidant activity than glucoside (arbutin) [21]. The rare report of the galactosidase-mediated glycosylation of phenolic compounds might be related to the low nucleophilicity of phenolic hydroxyl groups which result in the difficulty in the glycosylation by the enzymes in one aspect.

In this work, the preference of β-galactosidase from *L*. *bulgaricus* L3 toward the phenolic hydroxyl groups was improved through site-directed mutagenesis of the enzyme. The acceptor substrate range of the enzyme was broadened at the same time. The W980 residue that was presumed to be involved in substrate specificity was subjected to saturation mutagenesis. One mutation of tryptophan into phenylalanine changed the specificity of acceptor substrates, resulting in significantly higher preference toward phenolic acceptors. As a result, a series of novel phenolic galactosides were obtained by the β-galactosidase for the first time. This was a breakthrough in the enzymatic galactosylation of the challenging phenolic compounds of great values.

## Materials and Methods

### Strains and plasmids

*Escherichia coli* DH5α [F^-^ endA1 glnV44 thi^-1^ recA1 relA1 gyrA96 deoR nupG Φ80dlacZΔM15 Δ(lacZYA-argF)U169, hsdR17(rK^-^ mK^+^), λ^-^] and BL21(DE3) {F^-^ompT gal dcm lon hsdSB(rB^-^ mB^-^) λ(DE3 [lacI lacUV5-T7 gene 1 ind1 sam7 nin5]} were kept in the lab and cultured in LB medium containing 10 g of peptone, 5 g of yeast extract and 5 g of NaCl in 1,000 ml of water (pH 7.5). The solid medium additionally included 15 g/L agar. The recombinant strains carrying pET-21b (+) (Invitrogen) was cultured in LB medium plus ampicillin (100 μg/mL). The β-galactosidase gene (*bga*L3) from *L*. *bulgaricus* L3 (GenBank No. EU734748.1) was inserted into the pET-21b vector (pET-21b-bga) in the previous report [22].

### Sequence analysis and protein modeling of the β-galactosidase from *L*. *bulgaricus* L3 (BgaL3)

The amino-acid sequences of the β-galactosidases from various sources were aligned using Clustalw2 (http://www.ebi.ac.uk/Tools/msa/clustalw2/). Homology modeling of the BgaL3 was carried out using Phyre2 (http://www.sbg.bio.ic.ac.uk/phyre2/html/page.cgi?id=index). Images of the model were displayed by the software Pymol-1.4.1.

### Mutagenesis of β-galactosidase

Site-directed mutagenesis was performed by using Easy Mutagenesis kit (TransGenBiotech, China). The forward and reverse primers (5’-CGGGGATGACTCC**NNN**GGGCAGAAGGTCCA-3’ and 5’-**NNN**GGAGTCATCCCCGCCGACCCCCATCTG-3’) were designed to replace the W980 residue. The nucleotide sequences of **NNN** in primers for 19 amino-acid substitutions were listed in S1 Table. Each of the substitutions was carried out using the specific primers with the pET-21b-bga vector as template. PCR reactions were performed in the presence of TaKaRa LA Taq polymerase, following the procedures of 5 min at 94°C, 20 cycles of 30 s at 94°C, 30 s at 55°C, 7 min at 72°C, and a final 10 min at 72°C. The amplified fragments were treated with *Dpn* I enzyme (TaKaRa) for the template removal, and then were transformed into *E*. *coli* DH5α. The mutant plasmids were extracted from *E*. *coli* and sequenced to confirm the above mutations in the β-galactosidase gene.

### Analysis of the hydrolysis and transglycosylation activity of 19 mutants

Clones of the correct DH5α mutants were inoculated into 50 mL LB and grown at 37°C for 12 h. Then, the culture was centrifuged at 12000 rpm for 5 min. The resulting cell precipitate was resuspended in 1 mL of 50 mM phosphate buffer (pH 7.0) and freeze-thawed as crude enzyme directly for screening, since DH5α cells containing empty plasmid showed no β-galactosidase activity. The cells producing the wild-type enzyme were included as a reference at the same time. The hydrolysis activity toward *o*-nitrophenyl-β-D-galactopyranoside (*o*NPGal) was determined according to the enzyme assay described below. Transglycosylation reactions of the mutants toward phenolic acceptors were performed using hydroquinone as the screening acceptor. The reactions were carried out by incubation of 70 μL crude enzyme with 20 μL lactose (finally at 200 mM) and 10 μL of hydroquinone (finally at 100 mM) at 37°C for 1 h. Self-condensation reactions were performed by incubating the crude enzyme (70 μL) with 30 μL lactose (finally at 300 mM) at 37°C for 1 h. Reactions were stopped by heating at 100°C for 5 min and analyzed by TLC as described below.

### Preparation of the W980F enzyme

One mutant (W980F) with the highest transglycosylation activity toward hydroquinone was analyzed in detail. The recombinant plasmid carrying the mutation was extracted from *E*. *coli* DH5α and transformed into the strain of BL21(DE3). The transformant was grown in 30 mL of LB medium containing ampicillin at 37°C for 12 h, and then transferred into 1 L of fresh medium in a 1:100 (v/v) ratio. The enzyme was induced by addition of isopropyl-1-thio-β-D-galactoside (IPTG) when the cell density reached 0.6–1.0 at 600 nm. After continuous cultivation for 3 to 5 h, cells were harvested and disrupted by ultrasonic treatment. The lysate was centrifuged at 12000 rpm for 30 min at 4°C and the enzyme was purified from the suspension by Ni^2+^ chelation chromatography (Qiagen, Germany). The wild-type enzyme was prepared with the same procedures.

### Enzyme and protein assays

The β-galactosidase activity was measured by adding 50 μL of enzyme solution to 450 μL of 2 mM *o*NPGal. The reaction was performed at 37°C for 10 min and then stopped by adding 1 mL of 500 mM Na_2_CO_3_. The amount of *o*-nitrophenol released was measured at 420 nm. One unit of enzyme activity (U) was defined as the amount of enzyme required to liberate 1 μmol of *o*-nitrophenol per minute under the assay conditions. The amount of protein was quantified by the method of Lowry with bovine serum albumin as the standard.

### Characterization of mutant and wild-type BgaL3

Kinetic constants of mutant and wild-typle BgaL3 were estimated at 37°C and the data was processed using non-linear least square fit. Various concentrations of *o*NPGal (0.5 to 50 mM) and lactose (5 to 500 mM) were used to determine the kinetic constants, respectively. Reactions for *o*NPGal were performed under the enzyme assay conditions as described above. While in the case of lactose, the released glucose was measured by a commercially available kit using the glucose oxidase reaction (Biosino Bio-technology and Science, China).

The effect of pH on the activity of mutant and wild-type BgaL3 was assayed by incubating the enzyme with 2 mM *o*NPGal in 30 mM broad-range buffers, containing 6.01 g/L citric acid, 3.89 g/L KH_2_PO_4_, 1.77 g/L boric acid, and 5.27 g/L barbitone and using NaOH to adjust the pH from 3 to 11. The pH stability was determined by measuring enzyme activity after incubation of the enzyme in the same pH range at 4°C for 24 h. The effect of temperature on the enzyme activity was tested at 30 to 60°C for 10 min. Thermal stability was studied by assessing enzyme activity after incubation at the above temperatures for 0.5 h. To determine the effects of some chemicals, enzyme activities were assayed in the presence of 1 mM metal salts or 10 mM additives. All assays were performed in triplicate.

### Transglycosylation reactions

The transglycosylation with the phenolic compounds were performed at 45°C for 45 min in 50 μL mixtures (pH 7) containing 0.2 μg of pure enzyme, 200 mM of lactose and 100 mM of each acceptor including phenol, hydroquinone, catechol, and pyrogallol. The control reactions followed the same conditions except for the use of inactivated enzyme. The incubation of the enzyme with the lactose donor also acted as a control reaction. All the reactions were terminated by heating at 100°C for 5 min. Glycoside products were detected by TLC and HPLC as described below.

### TLC and HPLC analysis

TLC was performed with Silica gel 60 F_254_ plates (Merck, Germany). The developing solvent was a mixture of *n*-butanol: ethanol: water (5: 3: 2, v/v/v). Sugars on the TLC plate were detected by spraying with a solution of 0.5% (w/v) 3,5-dihydroxytoluene dissolved in 20% (v/v) sulfuric acid and subsequent heating at 120°C for 5 min. HPLC was performed by Agilent 1200 series equipped with Agilent Zorbax carbohydrate analysis column (4.6 × 250 mm). The column temperature was maintained at 30°C. Samples were eluted with 85% (v/v) acetonitrile at a flow rate of 1.0 mL/min, through a refractive index detector (G1362A) for the sugar analysis or a UV detector (G1314B) at 254 nm for the analysis of phenolic derivatives.

### Purification of glycoside products

Ten milliliters of transglycosylation reactions were performed at 45°C for 45 min. The resulting glycosylated products were concentrated by vacuum freeze dehydration. Then, the samples were loaded on a Bio-Gel P2 column (1.5 × 100 cm) and subsequently subjected to HPLC through a preparative column of Waters Spherisorb NH_2_ (10 x 250 mm) for purification. The corresponding eluates were pooled and lyophilized to dry powder.

### MS and NMR analysis

Mass spectra were recorded on a Shimadzu LCMS-IT-TOF instrument (Kyoto, Japan) equipped with an ESI source in positive/negative ion mode at a resolution of 10,000 full width at half-maximum. ^1^H and ^13^C NMR spectra were recorded at 26°C with a Bruker DRX Advance-600 spectrometer (Bruker Biospin AG, Fallanden, Switzerland) at 600 MHz for ^1^H and 150 MHz for ^13^C. Chemical shifts were given in ppm downfield from internal TMS of D_2_O. Chemical shifts and coupling constants were calculated from a first-order analysis of the spectra. Assignments were fully supported by homo- and hetero- nuclear correlated 2D techniques, including COSY (correlation spectroscopy), HSQC (hetero-nuclear single quantum coherence) and HMBC (hetero-nuclear multiple band correlation) experiments following standard Bruker pulse programs.

## Results

### Determination of the possible amino-acid residue related to the substrate specificity

As for β-galactosidases, the mechanism of the enzyme from *E*. *coli* has been clearly elucidated with so many crystallographic structures such as PDB 1DP0, 1JYN and 1JYV. The lactose substrate is found to initially bind in a shallow mode of the enzyme, characterized by stacking on W999, which establishes interactions between the side chain of the tryptophan and the ring of the glucose group. Then the substrate moves into a deep mode, further into the active site [23]. All these suggested W999 play a key role in the initial substrate selection of the enzyme.

**Fig 1:**
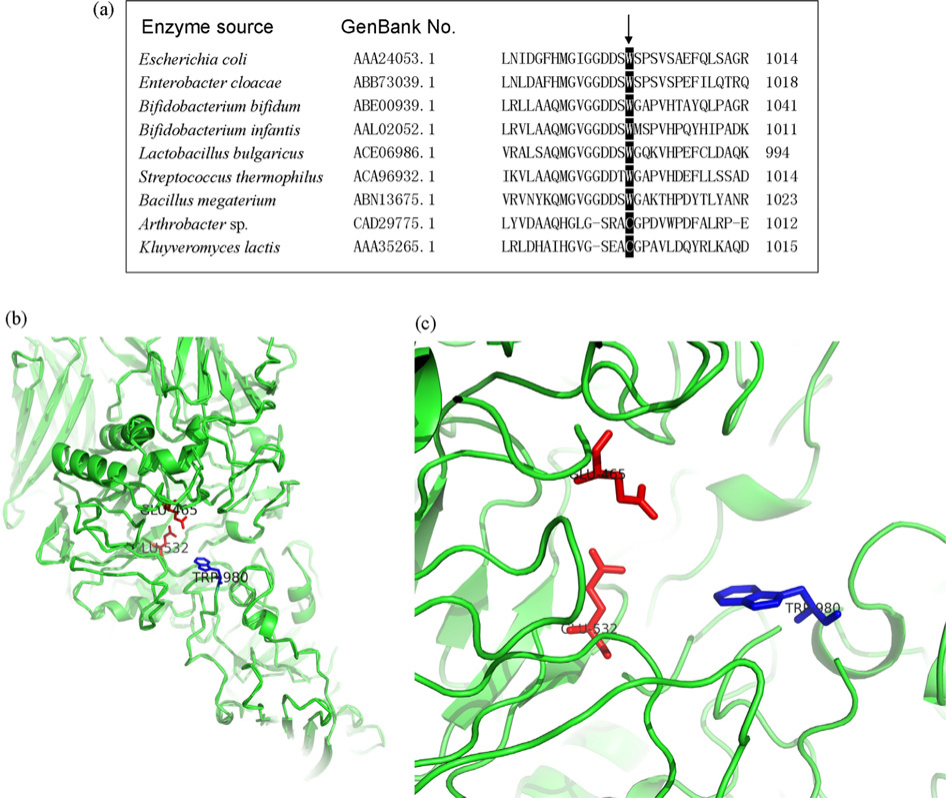
Multiple alignment of GH2 β-galactosidases (a) and the homology model of the whole BgaL3 (b) and its enlarged active center (c). The amino residues in the black boxing region are parallel to the *E*. *coli* W999. The putative catalytic residues of BgaL3, Glu465 and Glu532, locate at the bottom of the active center (in red), while the crucial residue involved in substrate specificity, W980, stands at the entrance of the active center (in blue).

Based on the amino-acid sequences, the β-galactosidases from *E*. *coli* and *Lactobacillus bulgaricus* L3 both belonged to glycosidase family (GH) 2. Fig. 1a shows the residue site corresponding to the W999 in *E*. *coli* was strictly conserved in most GH2 β-galactosidases, including the enzymes from *Escherichia*, *Enterobacter*, *Lactobacillus*, *Streptococcus* and *Bacillus*. In addition, the putative 3D-structure of BgaL3 revealed the residue (W980) paralleling to *E*. *coli* W999 located at the entrance of the active center while the putative catalytic residues (the acid-base Glu465 and the nucleophile Glu532) were present at the bottom (Fig. 1b and c), which was in a similar pattern to the β-galactosidase from *E*. *coli*. This theoretically confirmed the importance of W980 in the enzyme’s initial selection and action with substrate. Thus, the residue was selected for the subsequent mutagenesis.

### Site-saturation mutagenesis of BgaL3 and screening of mutants with improved transglycosylation activity on phenolic compound

**Fig 2:**
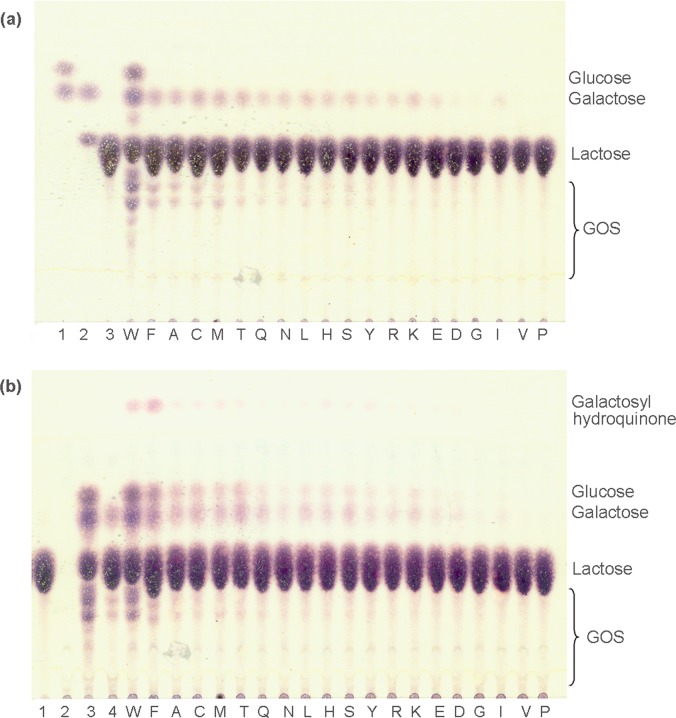
Transglycosylation reactions catalyzed by 19 mutants. (a) Lactose self-condensation. Lane 1, standard glucose plus galactose; lane 2, galactose plus lactose; lane 3, lactose. (b) Transglycosylation toward hydroquinone using lactose as donor. Lane 1, lactose; lane 2, hydroquinone (invisible in the plate); lane 3 and 4, reactions by the wild type and the W980F mutant only in the presence of the lactose, respectively. The capital letters ‘W’ to ‘P’ represent the results from crude enzymes bearing relevant amino-acid substitutions at the 980 site.

The W980 residue of BgaL3 was replaced by 19 amino acids through site-directed mutagenesis. All the resultant mutants displayed reduced hydrolysis activity toward the artificial substrate *o*NPGal as compared to the wild type. Among them, the hydrolysis activity of W980F showed the smallest decrease by ~58%. In Fig. 2a, all the mutants showed decreased activity toward lactose for both hydrolysis and transglycosylation. However, with the addition of the already-known phenolic acceptor, hydroquinone, the situation was significantly different for the W980F mutant. It produced hydroquinone glycoside in an obviously increased yield, along with simultaneous inhibition of lactose self-transglycosylation (Fig. 2b). The galactosylation of hydroquinone was confirmed by MS and NMR analysis (S1–S5 Figs.). Effects of the mutation on enzyme properties were further investigated.

### Preparation and characterization of the W980F enzyme

The W980F enzyme was expressed in *E*. *coli* BL21 (DE3) using IPTG as inducer. It was purified by affinity chromatography and subjected to biochemical assay. The kinetic parameters of the wild-type and mutant enzymes were shown in Table 1. The *K*_m_ values of W980 increased as compared with BgaL3, and the *K*_m_ for lactose rose much more significantly than that for *o*NPGal.

**Table 1:** Kinetic parameters of the wild-type BgaL3 and the mutant W980F.

Kinetic parameters	BgaL3		W980F	
	*o*NPGal	Lactose	*o*NPGal	Lactose
*K*_m_ (mM)	1.38	4.44	6.40	55.69
V_max_ (mmol min^-1^ mg^-1^)	6.53	0.33	0.64	0.42
*k*_cat_ (s^-1^)	1.25 × 10^4^	6.31 × 10^2^	1.24 × 10^3^	7.96 × 10^2^
*k*_cat_/*K*_m_ (mM^-1^ s^-1^)	9.06 × 10^3^	1.42 × 10^2^	1.94 × 10^2^	14.29

**Fig 3:**
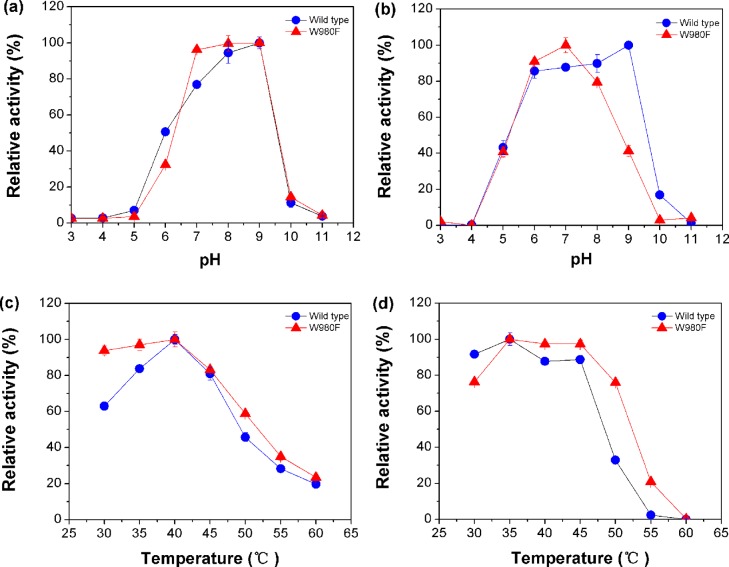
Effects of pH and temperature on the activity (a, c) and stability (b, d) of the wild-type and mutant enzymes.

The effects of pH and temperature on enzyme activity of W980F were similar to the wild type. As shown in Fig. 3a and b, both enzymes were highly active at pH 7.0 to 9.0 and stable between pH 6.0 and 8.0. The wild-type enzyme showed maximal stability at pH 9.0. In Fig. 3c and d, the two enzymes exhibited good activities at 40°C and stable at 35°C to 45°C. However, the mutant displayed tolerance for higher temperatures. It could keep 76% enzyme activity after incubation at 50°C for 0.5 h, whereas the wild type could only remain 33% activity under the same condition.

**Fig 4:**
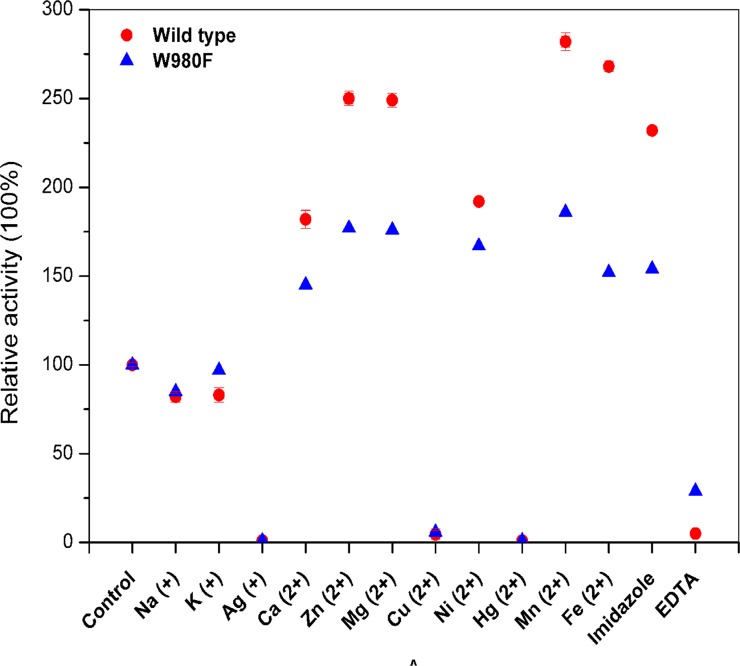
Effects of ions and additives on the activity of the wild-type and mutant enzymes.

The metal ions and additives showed consistent effects on both enzymes (Fig. 4). Hg^2+^, Cu^2+^ and Ag^+^ nearly completely inhibited activities of both enzymes, while Ca^2+^, Ni^2+^, imidazole, Mg^2+^, Zn^2+^, Fe^2+^ and Mn^2+^ obviously activated enzyme activities. Nevertheless, the activation and inhibition impact on the mutant enzyme became much slighter than that on the wild type. Additionally, EDTA nearly removed the activity of the wild type whereas the mutant enzyme could remain 29% activity. This indicated the mutant became less dependent on the ions for enzyme activity.

### Transglycosylation toward phenolic acceptors

**Fig 5:**
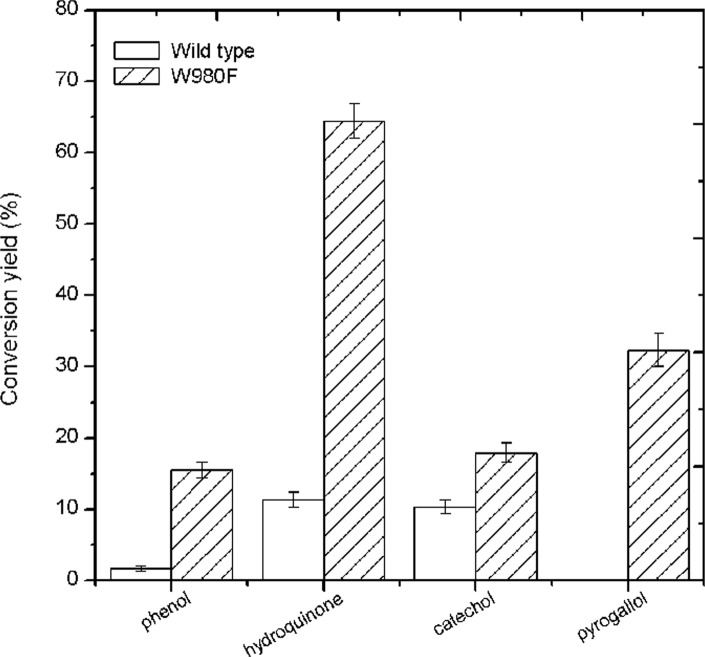
Tansglycosylation activity with various phenolic acceptors by the wild-type and mutant enzymes. Conversion yield of aromatic product (%): [product concentration (mM)/concentration of lactose used (mM)] × 100 [21].

As for the tested phenolic acceptors, both wild-type and mutant enzymes showed transglycosylation activity on simple monophenol and diphenol, including phenol, hydroquinone, and catechol. The mutant enzyme gave enhanced conversion yields of glycosides derived from these compounds by 13.8%, 53.1% and 7.6%, respectively, as compared with the wild type (Fig. 5). As for triphenol (i.e. pyrogallol), the wild-type enzyme showed no transferase activity, whereas the mutant enzyme could generate 32.3% yields of products from this difficult acceptor (Fig. 5).

**Fig 6:**
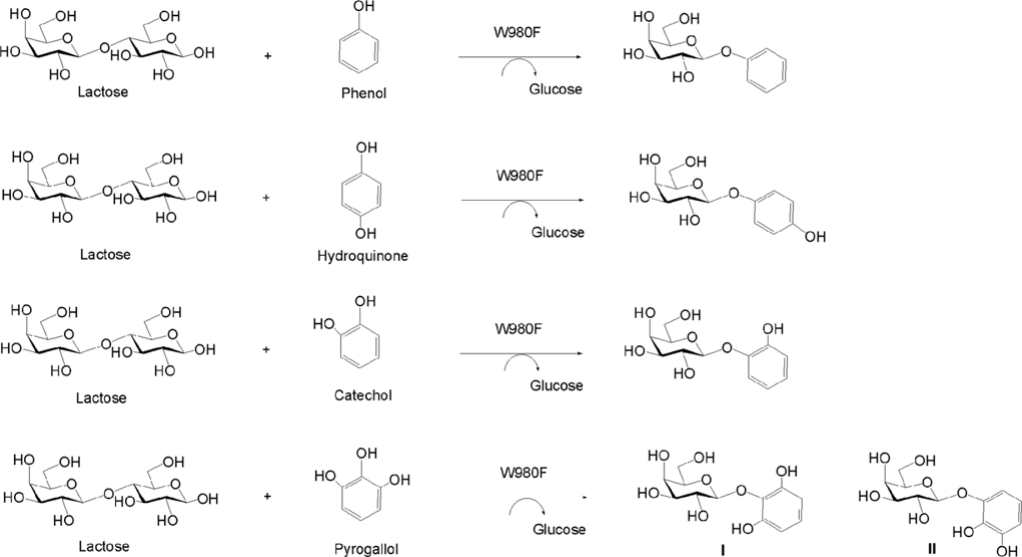
W980F-catalyzed glycosylation of phenolic compounds and chemical structures of the aromatic glycoside products.

All the newly produced glycosides were purified and the galactosylations were confirmed by MS analysis (S6 Fig.). The peaks of [M+H_2_O]^+^ ion at *m/z* 274.27 and [M+Na]^+^ at *m/z* 279.08 revealed the structure of phenol monogalactoside (*M*_*r*_ 256). The peak of [M+Na]^+^ at *m/z* 295.07 showed the presence of catechol monogalactoside (*M*_*r*_ 272). Signals of [M+Na]^+^ at *m/z* 311.07 characterized two pyrogallol monogalactosides (**I** and **II**) (*M*_*r*_ 288), respectively. The glycoside product from the phenol was further identified to be β-form galactoside based on the analysis of ^1^H NMR spectrum that revealed an anomeric signal at 4.87 ppm along with the coupling constant between H-1 and H-2 being 7.2 Hz (S7–S10 Figs.). Chemical structures of the novel glycosides derived from polyhydroxy phenolic acceptors were detailedly elucidated below and shown in Fig. 6.

In the spectra of catechol derivative, chemical shifts of the galactose protons located at *δ* 4.83 to 3.59 ppm while those of the phenolic ring were in the range of *δ* 7.02 to 6.78 ppm in ^1^H NMR (S11 Fig.). The H-1 of the sugar ring revealed a characteristic double peak at 4.83 ppm (*J* = 7.8 Hz), implying a β-configuration of the moiety. In ^13^C NMR spectrum, chemical shifts of the sugar carbons located at *δ* 101.6 to 60.6 ppm while those from *δ* 145.4 to 116.4 ppm belonged to the phenolic residue (S12 Fig.). The complete structural characterization was achieved using 2D-NMR analysis, including ^1^H-^1^H COSY, ^1^H-^13^C HSQC and HMBC experiments, to assign the chemical shifts and configurations (S13–S15 Figs.). As shown in HMBC, the cross peak was obviously found between C-1′ of the phenolic ring (*δ* 144.5) and H-1 of the sugar ring (*δ* 4.83), conforming the glycosidic linkage between the two residues. Thus, the structure data of catechol β-galactoside could be summarized as follows. ^1^H NMR (600 MHz, D_2_O): *δ* 7.02 (H-6′), 6.86 (H-5′), 6.81 (H-3′), 6.78 (H-4′), 4.83 (H-1), 3.82 (H-4), 3.69 (H-5), 3.65 (H-2), 3.61 (H-6), 3.59 (H-3). ^13^C NMR (150 MHz, D_2_O): *δ* 145.4 (C-2′), 144.5 (C-1′), 123.9 (C-5′), 121.0 (C-4′), 116.6 (C-6′), 116.4 (C-3′), 101.6 (C-1), 75.3 (C-2), 72.4 (C-3), 70.4 (C-5), 68.4 (C-4), 60.6 (C-6).

Two monoglycosides (**I** and **II**) were formed from pyrogallol by the mutant enzyme as analyzed by TLC and MS (S6 Fig.). In the ^1^H NMR of **I** and **II**, the anomeric H-1 peaks of sugar residues were present at 4.70 and 4.76 ppm, respectively (S16 and S21 Figs.). Both coupling constants were 7.8 Hz, confirming the β-linkage between the sugar and pyrogallol. Structures of the two compounds were further identified to be pyrogallol 1′*-O-*galactoside (**I**) and pyrogallol 2*-O-*galactoside (**II**) by 2D-NMR (S16–S25 Figs.). The relevant data were summarized as follows. For the compound **I**, ^1^H NMR (600 MHz, D_2_O): *δ* 6.81 (H-4′), 6.38 (H-3′, H-5′), 4.70 (H-1), 3.78 (H-4), 3.66 (H-2), 3.60 (H-5), 3.55 (H-3), 3.50 (H-6); ^13^C NMR (150 MHz, D_2_O): 149.4 (C-2′, C-6′), 132.1 (C-1′), 126.0 (C-4′), 108.5 (C-3′, C-5′), 104.4 (C-1), 75.5 (C-5), 72.4 (C-3), 71.0 (C-2), 68.4 (C-4), 60.7 (C-6). For the compound **II**, ^1^H NMR (600 MHz, D_2_O): *δ* 6.59 (H-4′), 6.56 (H-3′), 6.48 (H-5′), 4.76 (H-1), 3.77 (H-4), 3.65 (H-2), 3.59 (H-5), 3.56 (H-6), 3.54 (H-3); ^13^C NMR (150 MHz, D_2_O): *δ* 145.6 (C-2′), 144.9 (C-6′), 134.2 (C-1′), 120.0 (C-4′), 111.0 (C-5′), 108.2 (C-3′), 101.6 (C-1), 75.3 (C-5), 72.3 (C-3), 70.4 (C-2), 68.3 (C-4), 60.6 (C-6).

## Discussion

Currently, only a few natural glycosidases are discovered to be able to glycosylate phenolic compounds [18]. The low nucleophilicity of phenolic hydroxyl groups might account for the difficulty in their modification by enzymes, as compared with alcoholic hydroxyl nucleophiles that are easily glycosylated. At present, the known glycosidases with tolerance for phenolic acceptors catalyze glucose transfer in most cases. The α-amylase (EC 3.2.l.1) from *Bacillus subtilis* is able to glucosylate caffeic acid using maltopentaose as the donor [16]. The α-glucosidase (EC 3.2.1.20) from *Saccharomyces cerevisiae* can modify hydroquinone in the presence of maltose [18]. Similar enzymes from *Xanthomonas campestris* and *X*. *maltophilia* are able to transfer glucosyl from maltose to the catechin and eugenol [14, 24, 25].

In the previous work, the β-galactosidase from *Lactobacillus bulgaricus* L3 was successfully used for the synthesis of prebiotic galacto-oligosaccharides from lactose self-transglycosylation [4, 5]. Later, it was applied to transfer 6'-galactosyl from lactose to sucralose, generating a novel promising compound with potentially combined functions of prebiotics and sweeteners [22]. These suggested the enzyme was a wonderful tool for glycoside synthesis. Recently, the enzyme was found to tolerate phenolic acceptors besides hydroquinone, but the conversion yields were rather low (data not shown). This work enhanced the transglycosylation on phenolic acceptors and broadened the acceptor specificity by molecular evolution of the enzyme. The mutation was performed at the W980 residue that was predicted to be important for substrate selectivity via bioinformatics analysis. The saturation mutagenesis was performed via individual replacement of tryptophan by other amino acids at the same time, resulting in 19 mutants for screening. This operation not only ensured the entirely saturated mutation but also avoided the laborious screening work.

A substitution of tryptophan with phenylalanine at 980 site enhanced transglycosylation activity toward simple hydroquinone. The mutant phenylalanine and the native tryptophan at the 980 site both contain aromatic structures, but they show slight difference in the side chains. The former bears a small benzene ring while the latter contains a slightly bigger ring of indole. The shorter side chains of phenylalanine might reduce the steric hindrance of the enzyme to hold phenolic substrates. Also it might facilitate the stacking action between phenylalanine and phenolic substrates, which would help the substrate enter the active center. Additionally, the catalytic efficiency (based on *k*_cat_/*K*_m_) of W980F for the hydrolysis of *o*NPGal reduced about 46 fold as compared with the wild-type enzyme, which was in agreement with the phenomena that the mutant enzyme nearly showed no hydrolysis activity toward various derivatives from phenolic acceptors, thus resulting in accumulation of the products in high yields.

In the transglycosylation reactions for phenolic acceptors, the self-condensations of lactose by the mutant enzyme were all obviously reduced, thus resulting in a half and more decreases of galacto-oligosaccharides yields (data not shown). Self-transglycosylation of sugar donors usually occurred in glycosidase-mediated synthesis. Such side reactions would have a negative effect on product yields and should be controlled for a general applicability of glycosidase in synthesis. Tran et al. had introduced one mutation (A221W) in the β-glycosidase from *Thermus thermophilus*, preventing the donor-self-condensation while remaining the condensation at a similar level [26]. Also in this work, a single mutation of the enzyme (W980F) inhibited the self-condensation without any harmful influence on its transfer of galactosyl to phenolic acceptors. The *K*_m_ value of the mutant enzyme for lactose displayed an about 12-fold increase, suggesting apparently decreased affinity toward the substrate.

Using the W980F mutant, a series of novel phenolic galactosides were achieved through glycosidase-mediated reactions for the first time. Except for hydroquinone, the phenol, catechol and pyrogallol were all newly discovered acceptors for β-galactosidase and they were modified by glycosidase for the first time. This wok was actually a breakthrough in the galactosylation of the difficult or unusual acceptors by glycosidases.

## Supporting Information

S1 Fig MS spectrum of the hydroquinone galactoside formed by the W980F enzyme. (TIF)

S2 Fig ^1^H NMR spectrum of the hydroquinone galactoside formed by the W980F enzyme. (TIF)

S3 Fig ^13^C NMR spectrum of the hydroquinone galactoside formed by the W980F enzyme. (TIF)

S4 Fig COSY spectrum of the hydroquinone galactoside formed by the W980F enzyme. (TIF)

S5 Fig HSQC spectrum of the hydroquinone galactoside formed by the W980F enzyme. (TIF)

S6 Fig MS analysis of the newly produced glycosides by the W980F enzyme. (a) phenol monogalactoside (*M*_*r*_ 256); (b) catechol monogalactoside (*M*_*r*_ 272); (c) pyrogallol monogalactoside **I** (*M*_*r*_ 288); (d) pyrogallol monogalactoside **II** (*M*_*r*_ 288).
(TIF)

S7 Fig ^1^H NMR spectrum of the phenol galactoside formed by the W980F enzyme. (TIF)

S8 Fig ^13^C NMR spectrum of the phenol galactoside formed by the W980F enzyme. (TIF)

S9 Fig COSY spectrum of the phenol galactoside formed by the W980F enzyme. (TIF)

S10 Fig HSQC spectrum of the phenol galactoside formed by the W980F enzyme. (TIF)

S11 Fig ^1^H NMR spectrum of the catechol galactoside formed by the W980F enzyme. (TIF)

S12 Fig ^13^C NMR spectrum of the catechol galactoside formed by the W980F enzyme. (TIF)

S13 Fig COSY spectrum of the catechol galactoside formed by the W980F enzyme. (TIF)

S14 Fig HSQC spectrum of the catechol galactoside formed by the W980F enzyme. (TIF)

S15 Fig HMBC spectrum of the catechol galactoside formed by the W980F enzyme. (TIF)

S16 Fig ^1^H NMR spectrum of the pyrogallol galactosides I formed by the W980F enzyme. (TIF)

S17 Fig ^13^C NMR spectrum of the pyrogallol galactosides I formed by the W980F enzyme. (TIF)

S18 Fig COSY spectrum of the pyrogallol galactosides I formed by the W980F enzyme. (TIF)

S19 Fig HSQC spectrum of the pyrogallol galactosides I formed by the W980F enzyme. (TIF)

S20 Fig HMBC spectrum of the pyrogallol galactosides I formed by the W980F enzyme. (TIF)

S21 Fig ^1^H NMR spectrum of the pyrogallol galactosides II formed by the W980F enzyme. (TIF)

S22 Fig ^13^C NMR spectrum of the pyrogallol galactosides II formed by the W980F enzyme. (TIF)

S23 Fig COSY spectrum of the pyrogallol galactosides II formed by the W980F enzyme. (TIF)

S24 Fig HSQC spectrum of the pyrogallol galactosides II formed by the W980F enzyme. (TIF)

S25 Fig HMBC spectrum of the pyrogallol galactosides II formed by the W980F enzyme. (TIF)

S1 Table Primers used in site-directed mutagenesis. (DOC)
