# Supplementary figures and images for: Glycosylation of Phenolic Compounds by the Site-Mutated β-Galactosidase from Lactobacillus bulgaricus L3
Source: PLoS One. 2015 Mar 24;10(3):e0121445. doi: 10.1371/journal.pone.0121445 (PMC4372403; doi:10.1371/journal.pone.0121445)

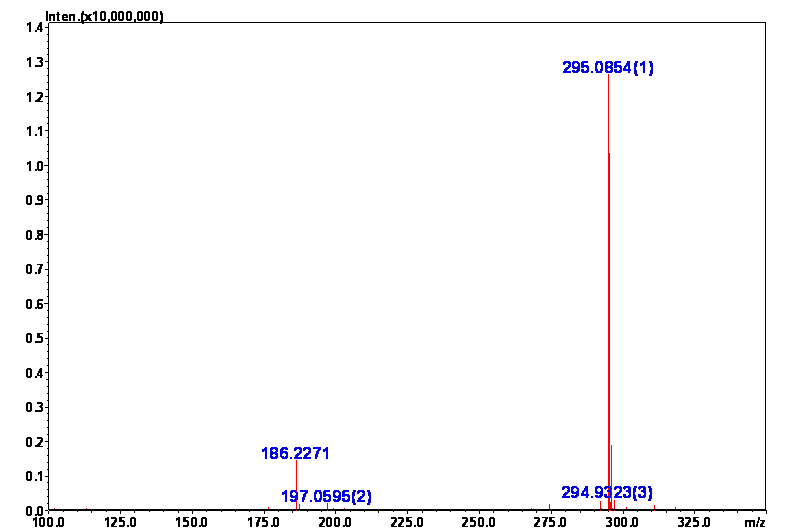

Supplement: S1 Fig — (TIF) [file pone.0121445.s001.tif]

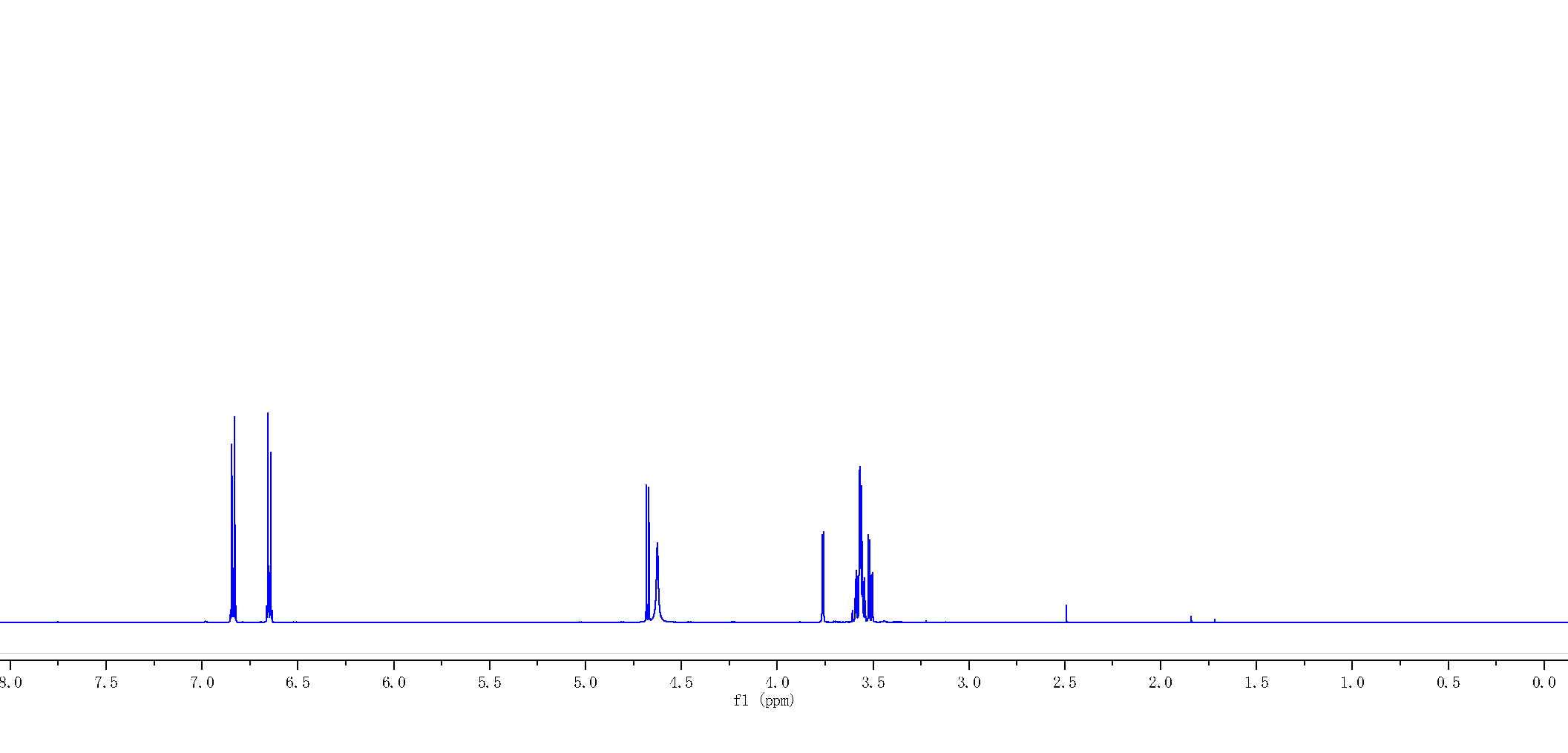

Supplement: S2 Fig — (TIF) [file pone.0121445.s002.tif]

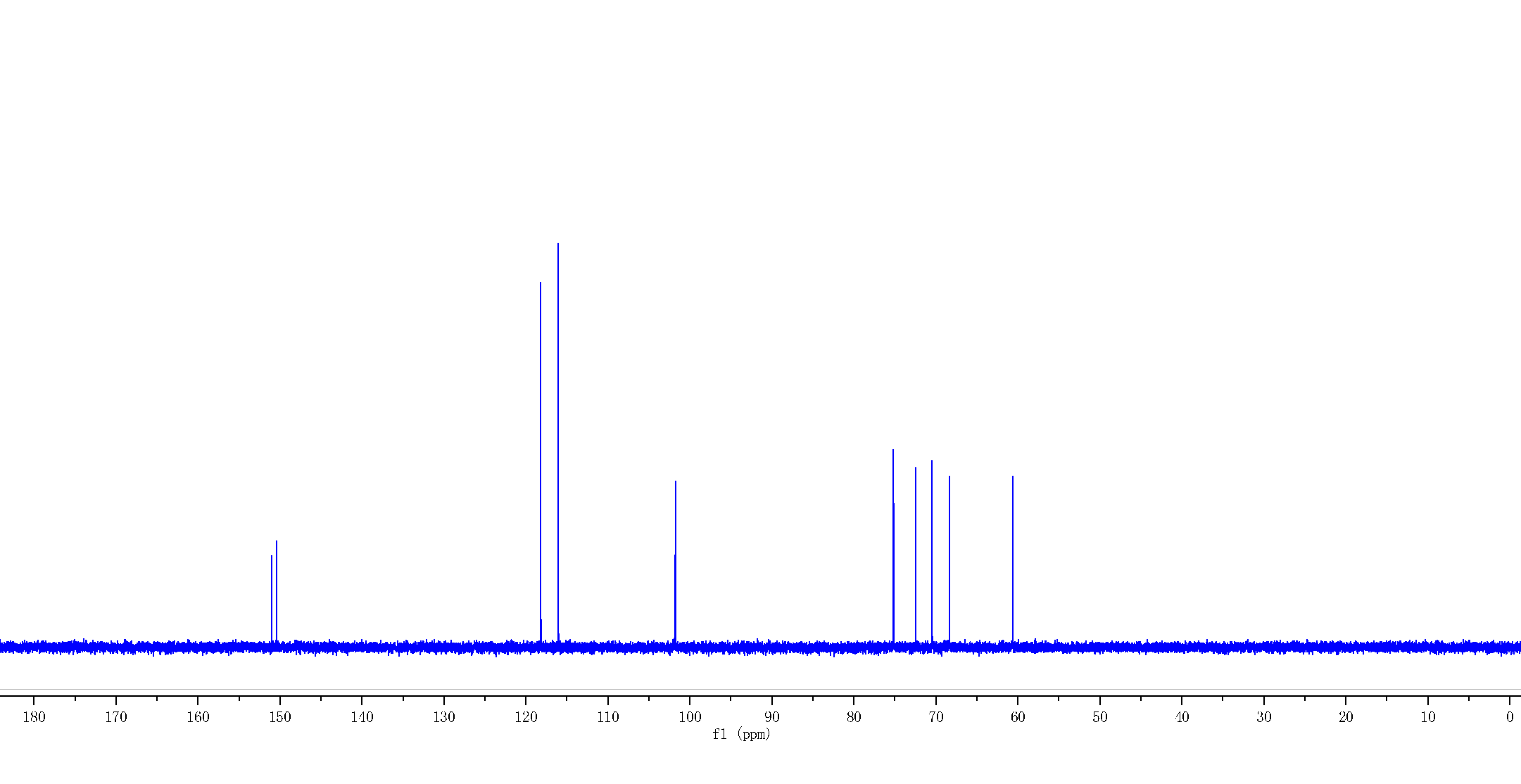

Supplement: S3 Fig — (TIF) [file pone.0121445.s003.tif]

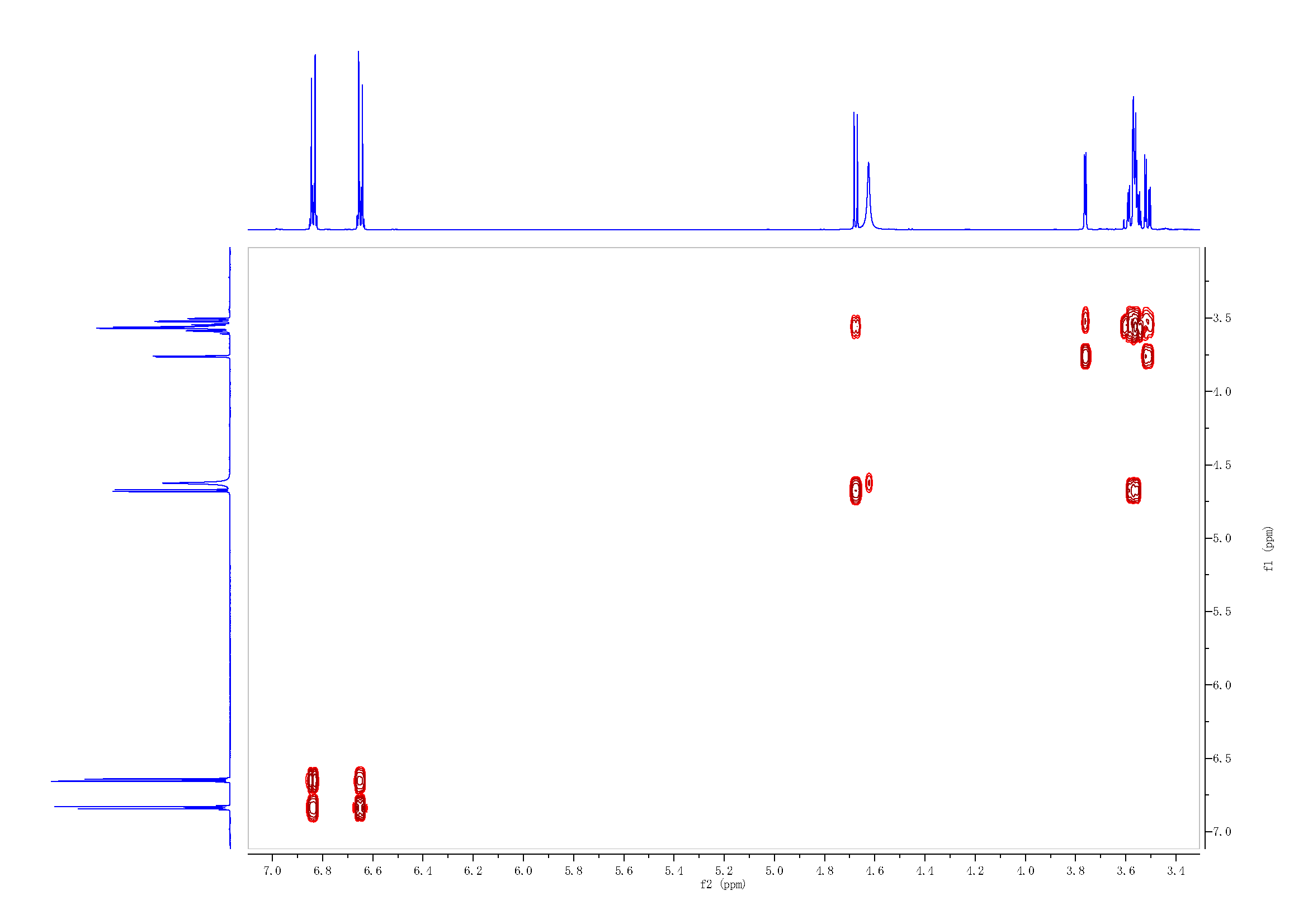

Supplement: S4 Fig — (TIF) [file pone.0121445.s004.tif]

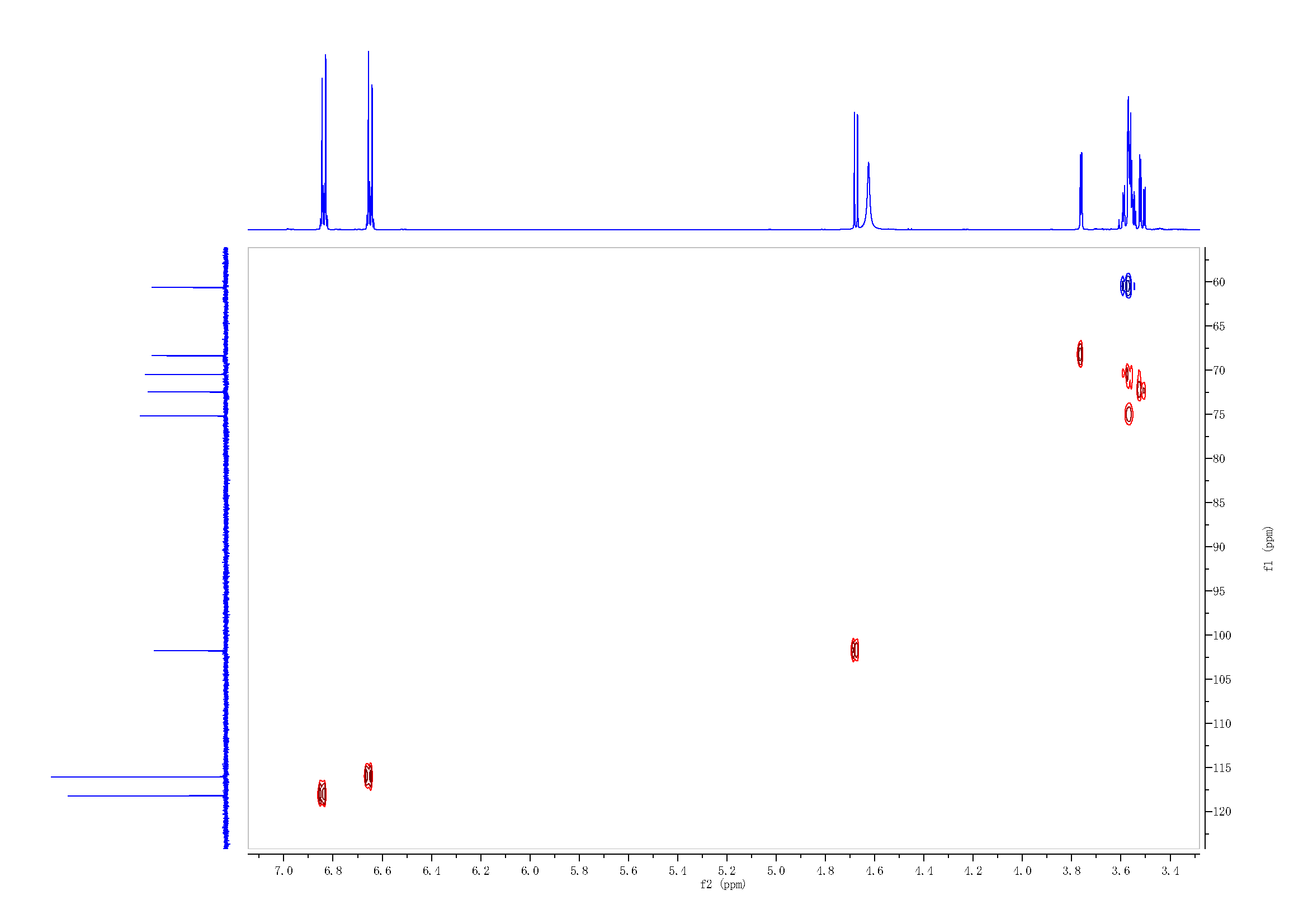

Supplement: S5 Fig — (TIF) [file pone.0121445.s005.tif]

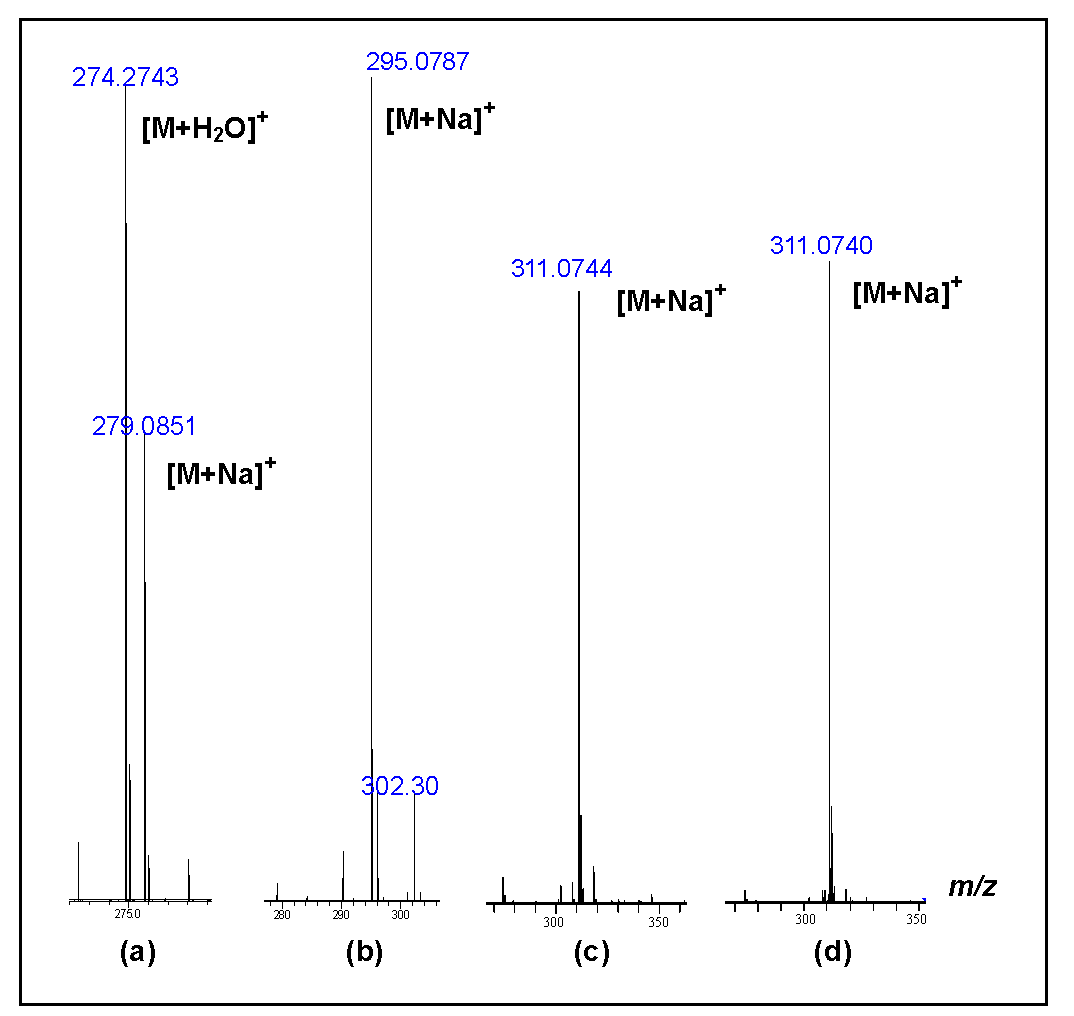

Supplement: S6 Fig — (a) phenol monogalactoside (M r 256); (b) catechol monogalactoside (M r 272); (c) pyrogallol monogalactoside I (M r 288); (d) pyrogallol monogalactoside II (M r 288). (TIF) [file pone.0121445.s006.tif]

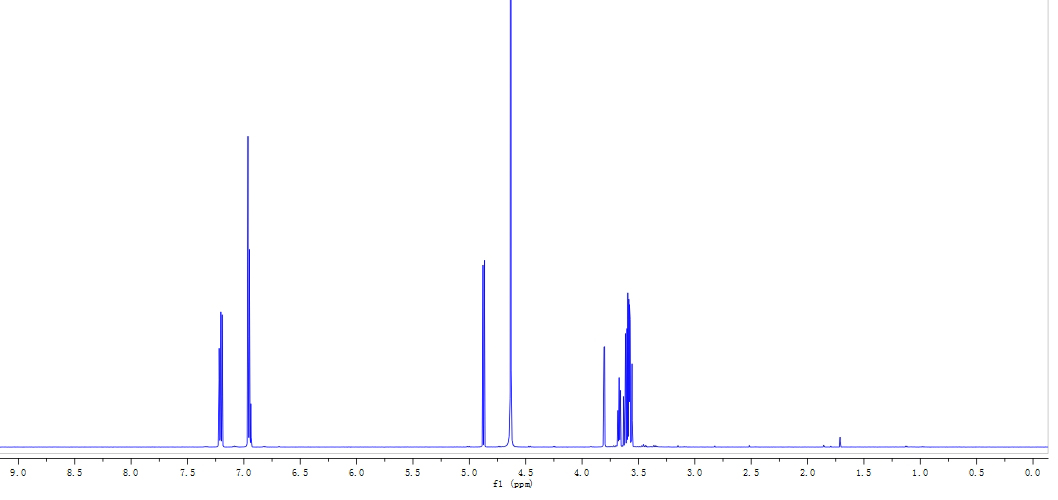

Supplement: S7 Fig — (TIF) [file pone.0121445.s007.tif]

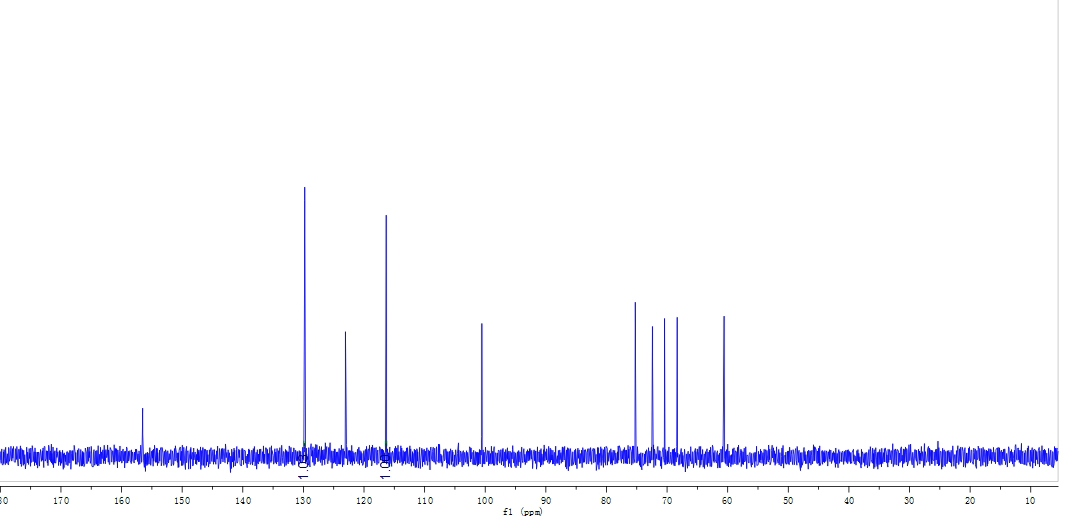

Supplement: S8 Fig — (TIF) [file pone.0121445.s008.tif]

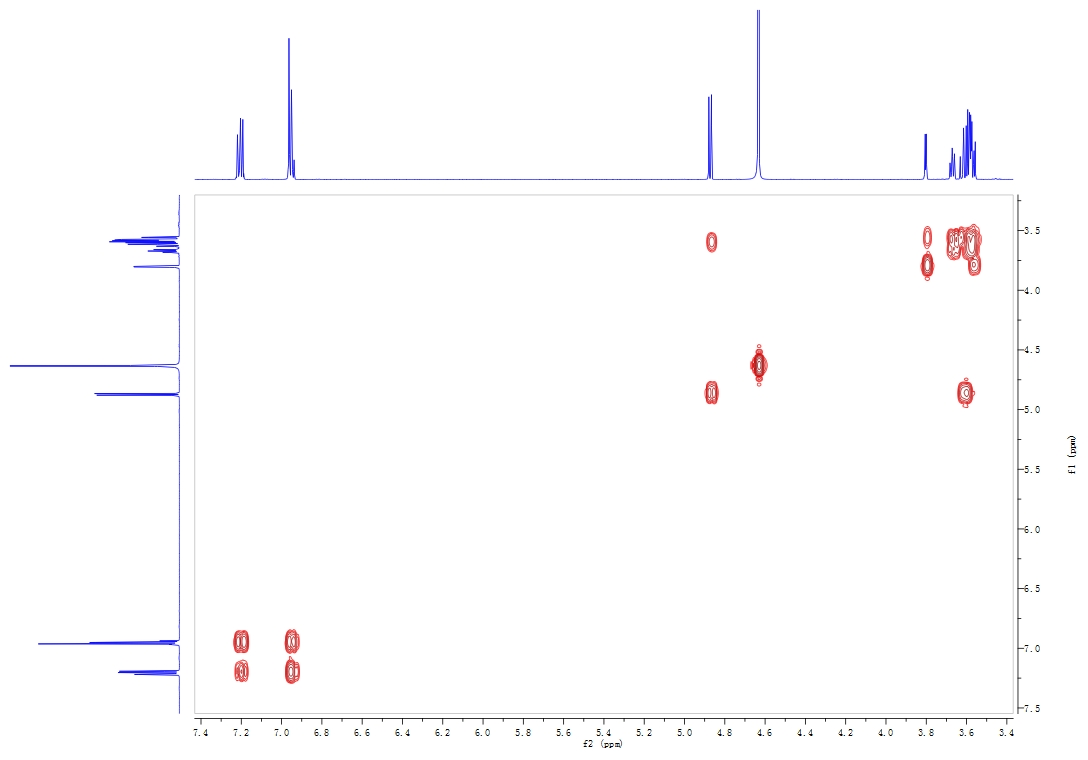

Supplement: S9 Fig — (TIF) [file pone.0121445.s009.tif]

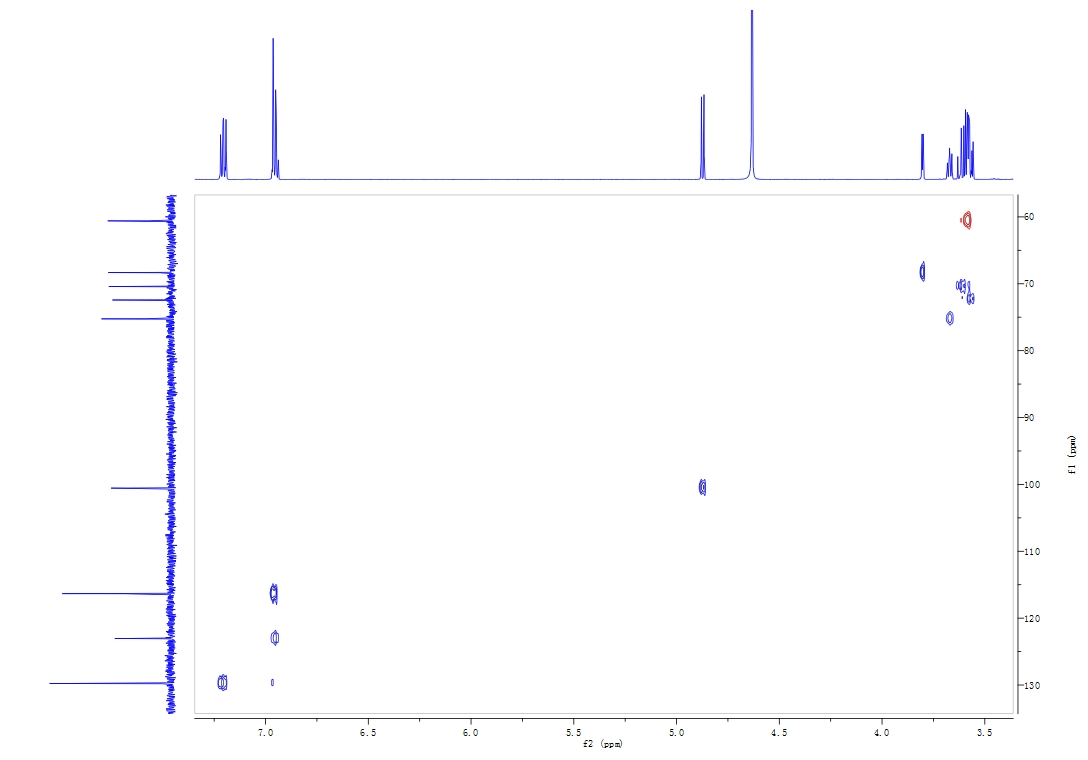

Supplement: S10 Fig — (TIF) [file pone.0121445.s010.tif]

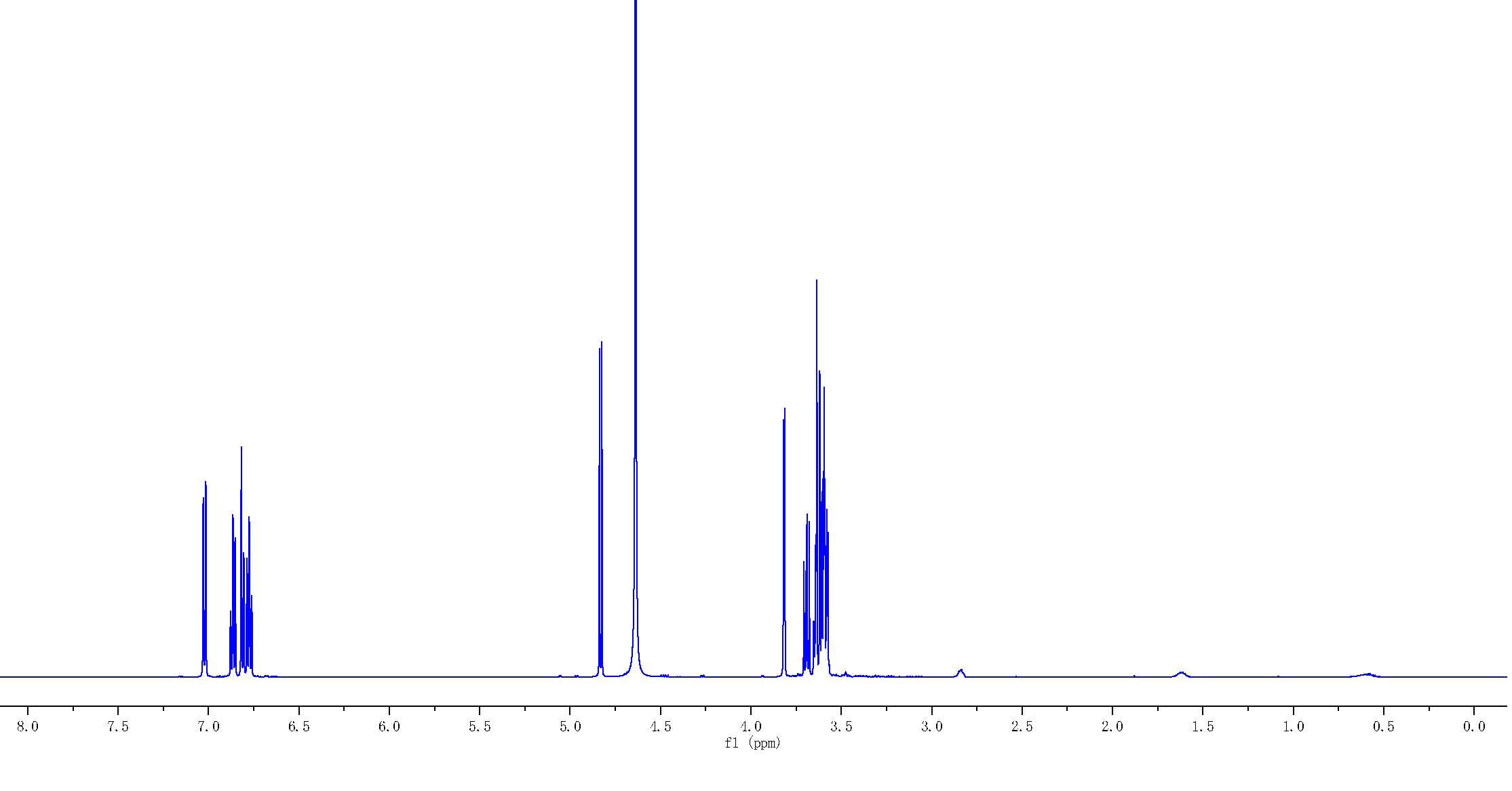

Supplement: S11 Fig — (TIF) [file pone.0121445.s011.tif]

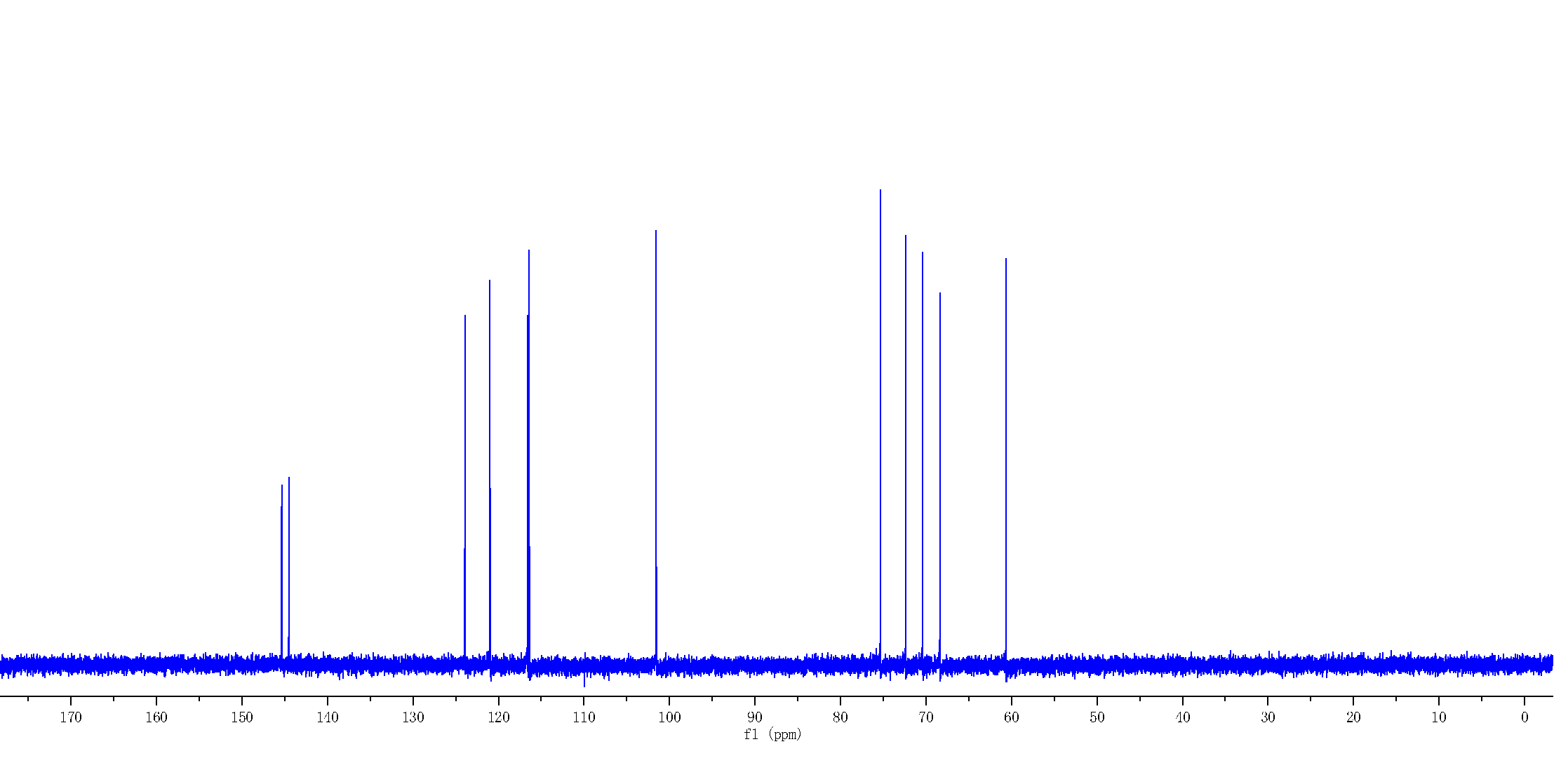

Supplement: S12 Fig — (TIF) [file pone.0121445.s012.tif]

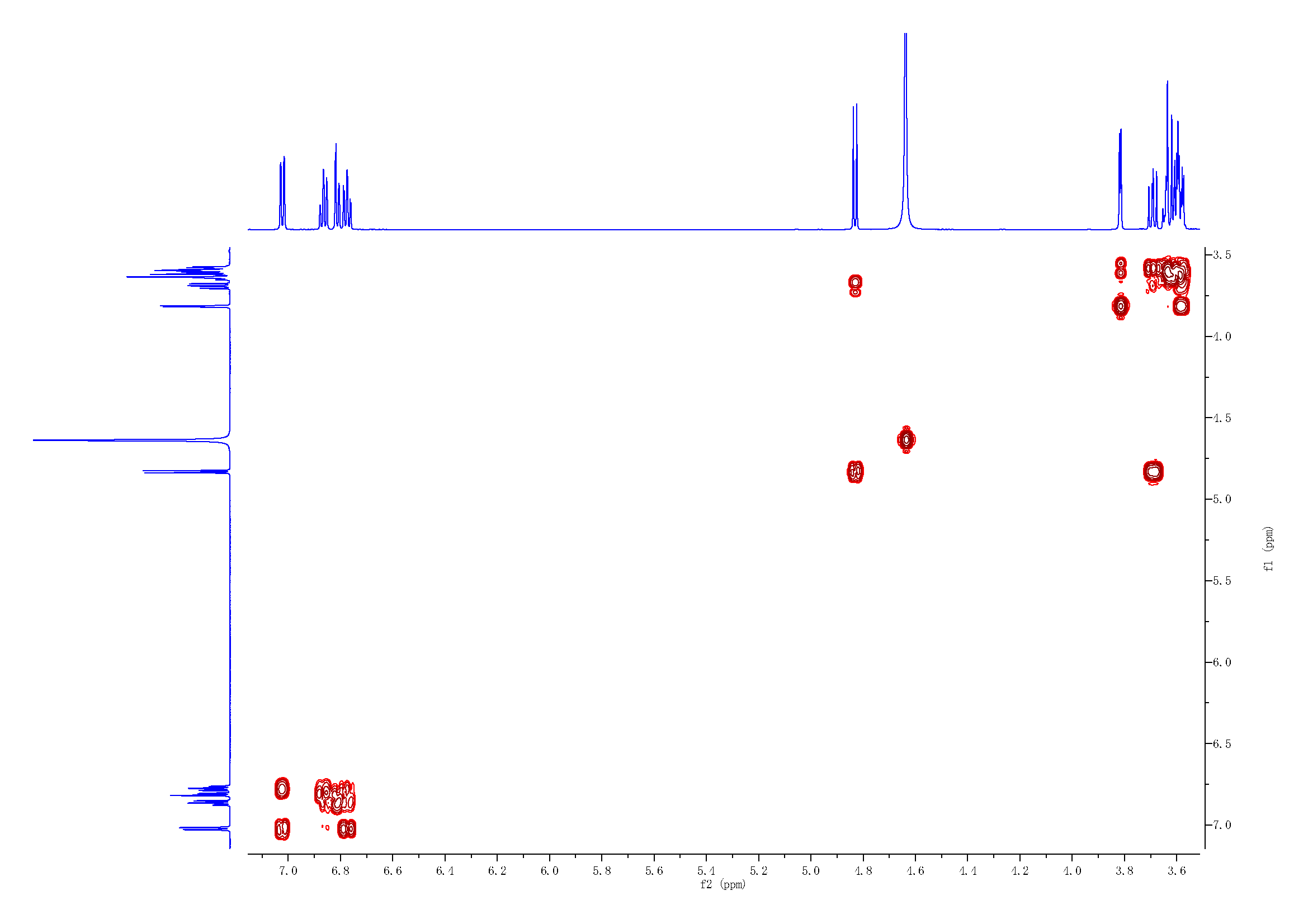

Supplement: S13 Fig — (TIF) [file pone.0121445.s013.tif]

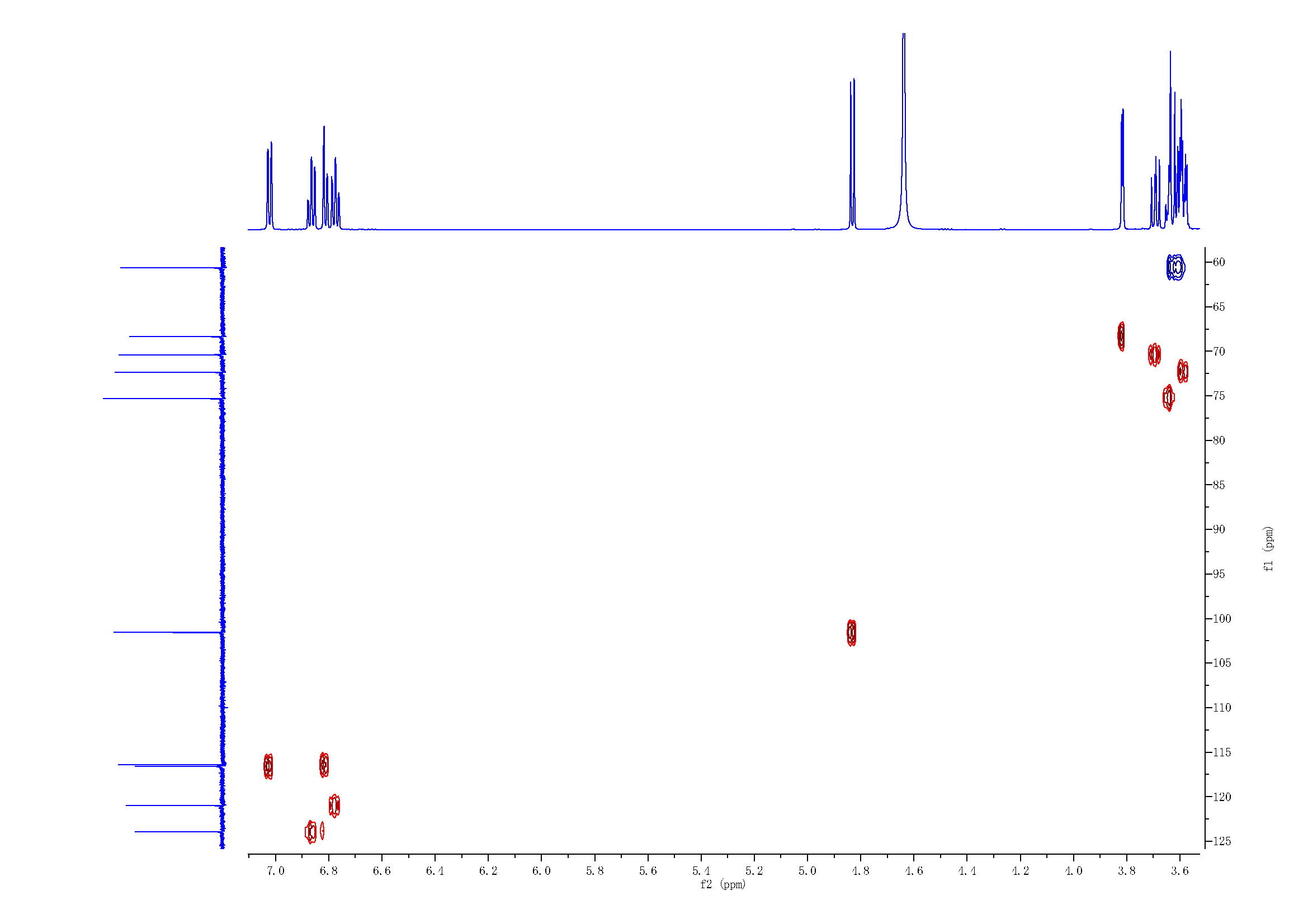

Supplement: S14 Fig — (TIF) [file pone.0121445.s014.tif]

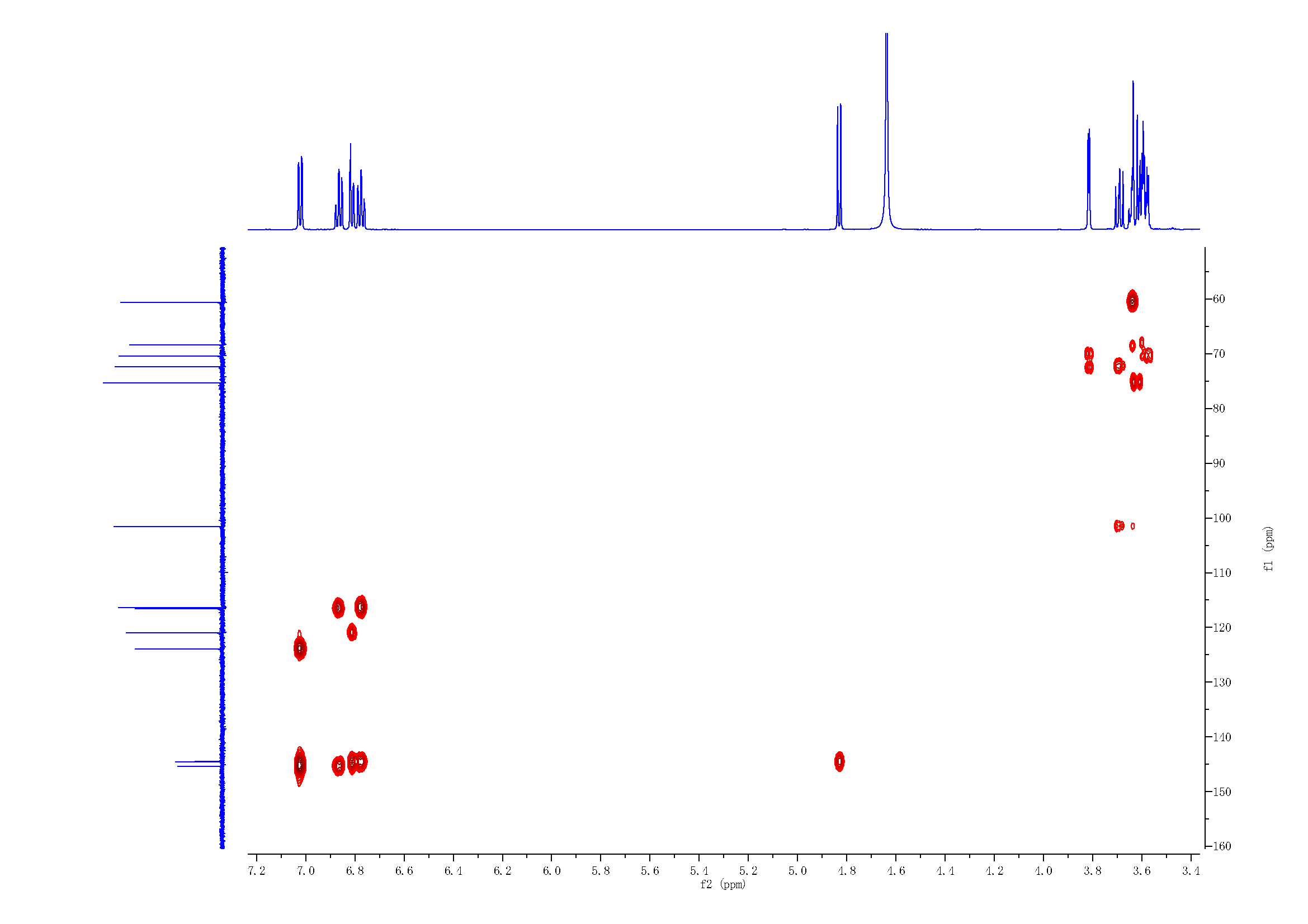

Supplement: S15 Fig — (TIF) [file pone.0121445.s015.tif]

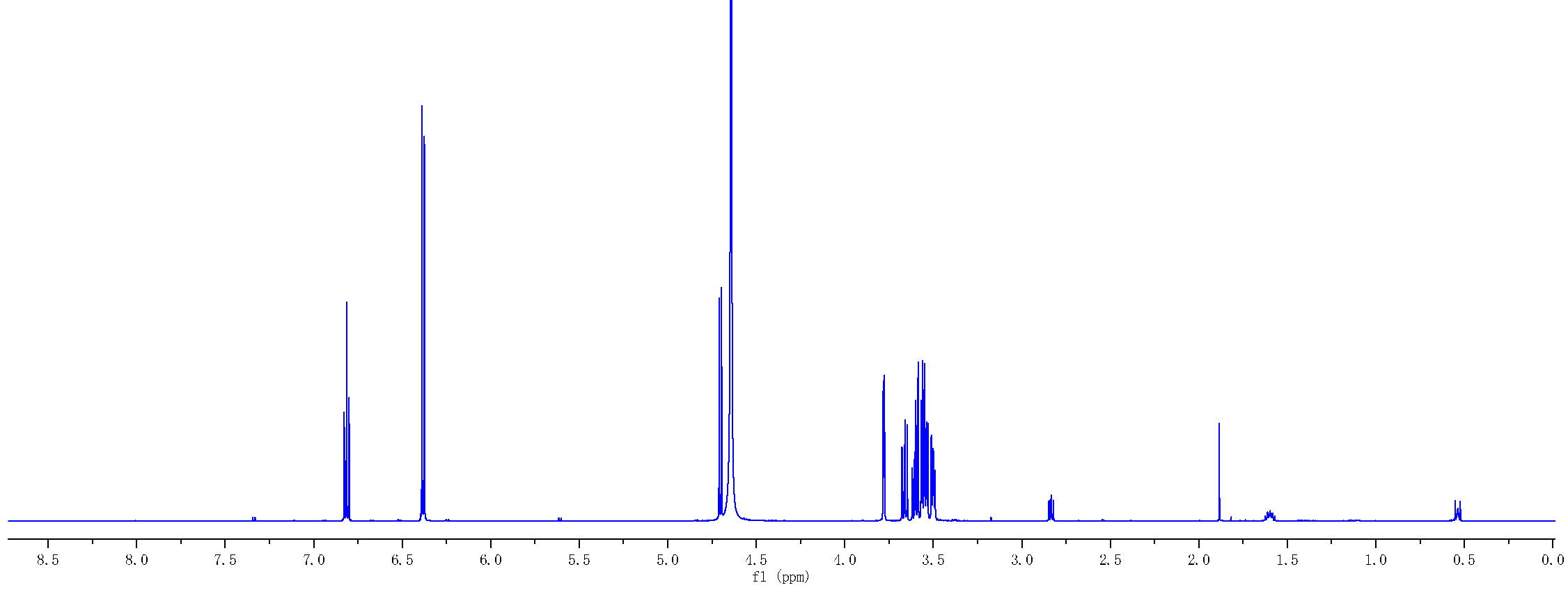

Supplement: S16 Fig — (TIF) [file pone.0121445.s016.tif]

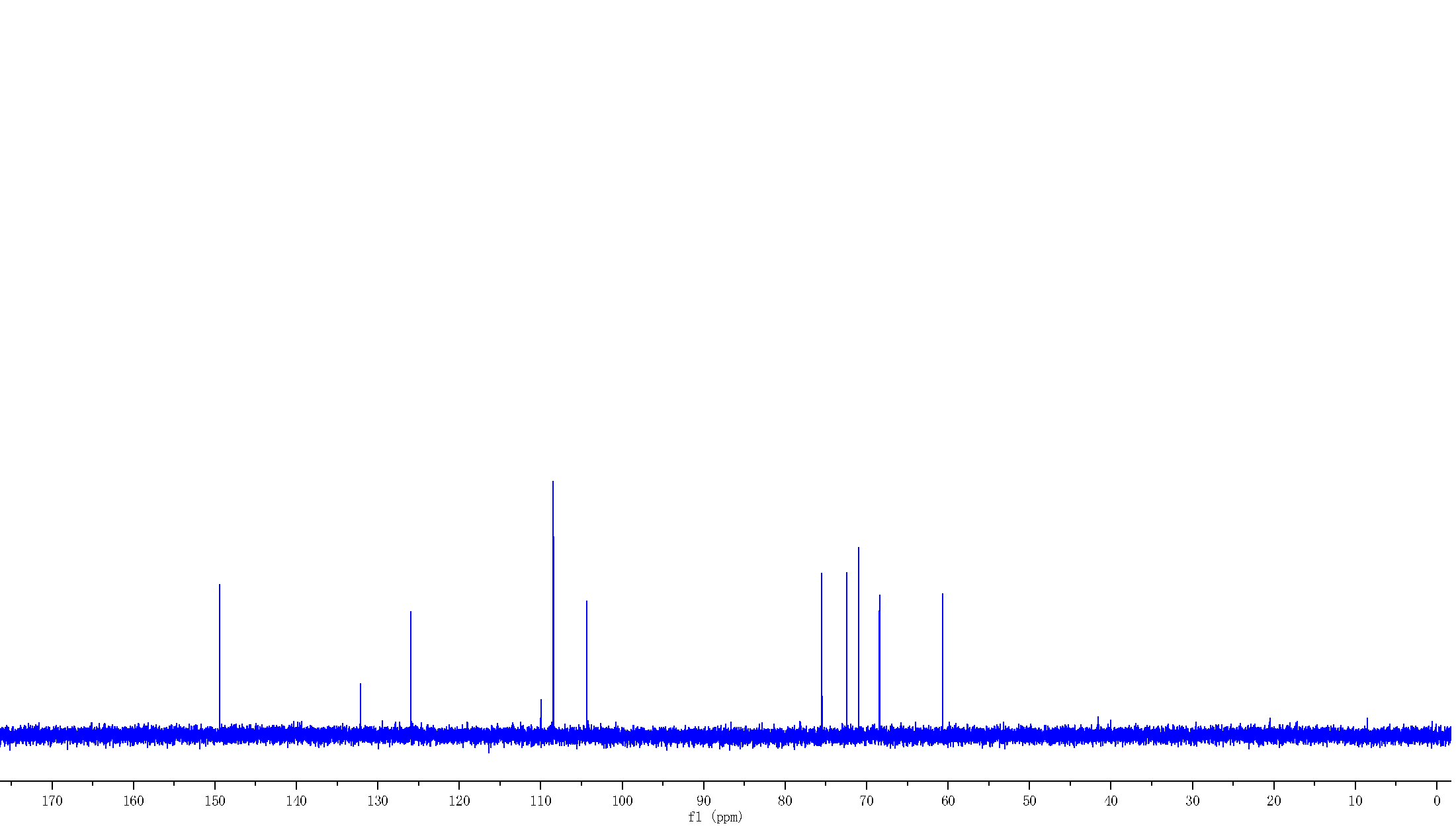

Supplement: S17 Fig — (TIF) [file pone.0121445.s017.tif]

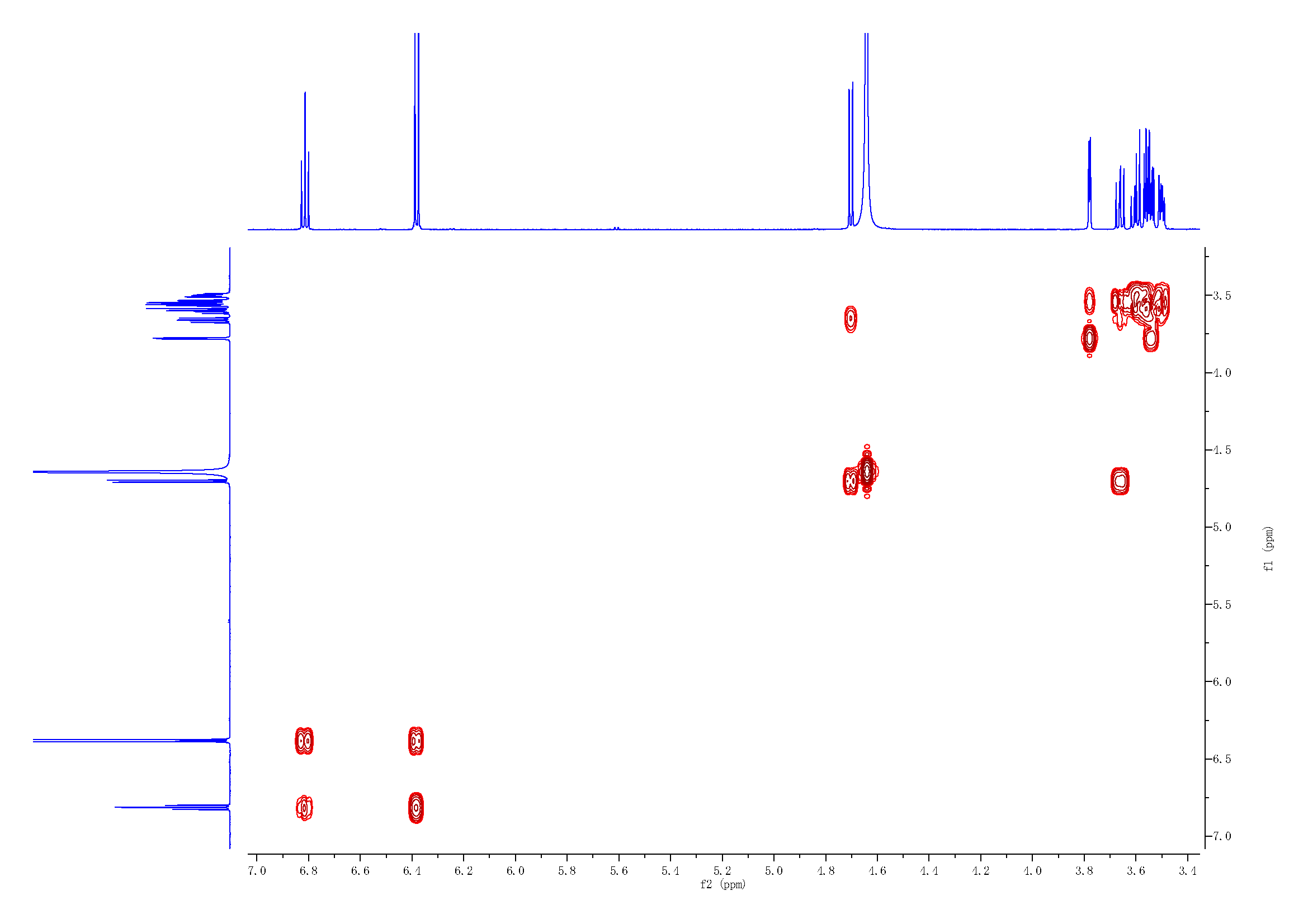

Supplement: S18 Fig — (TIF) [file pone.0121445.s018.tif]

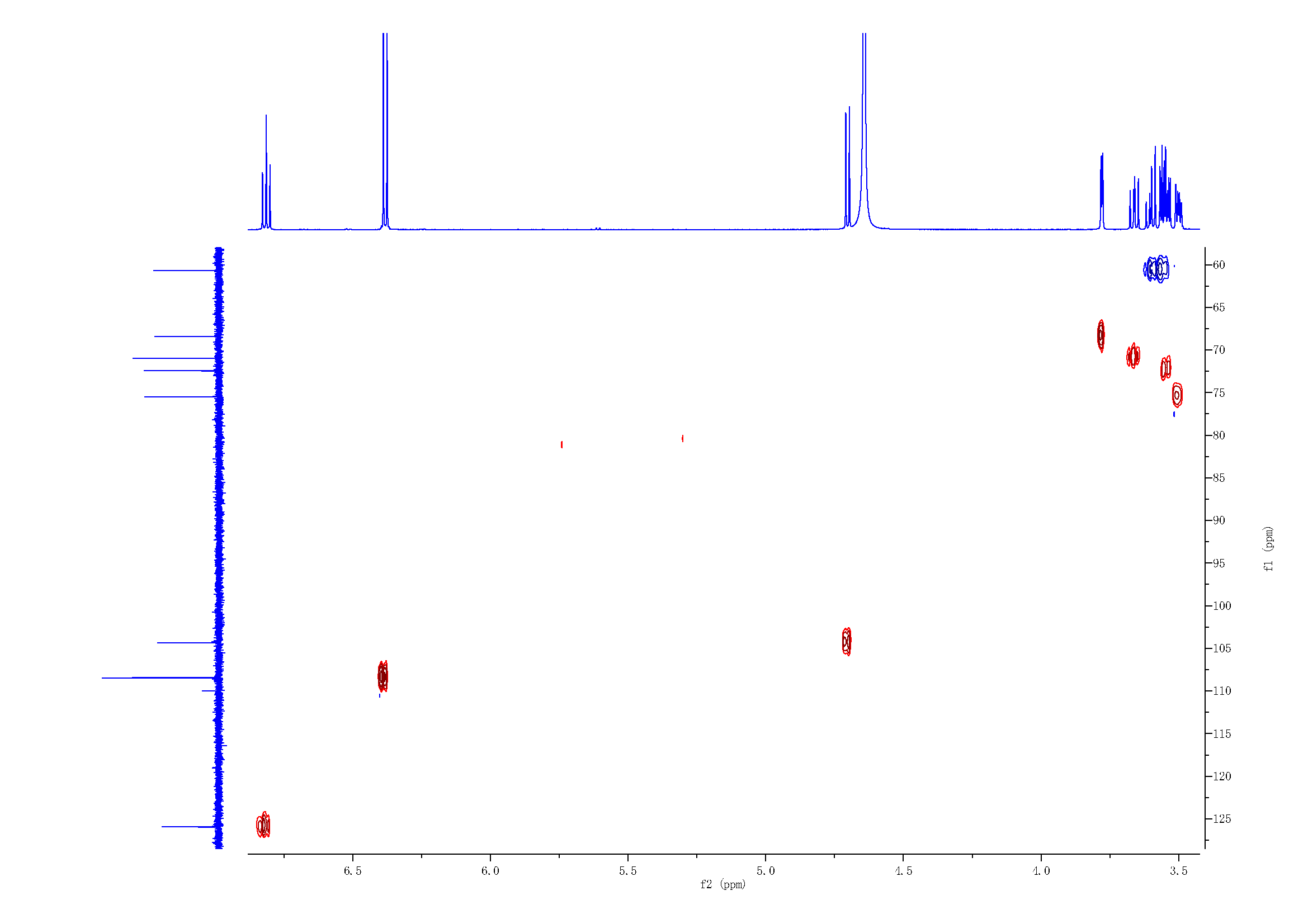

Supplement: S19 Fig — (TIF) [file pone.0121445.s019.tif]

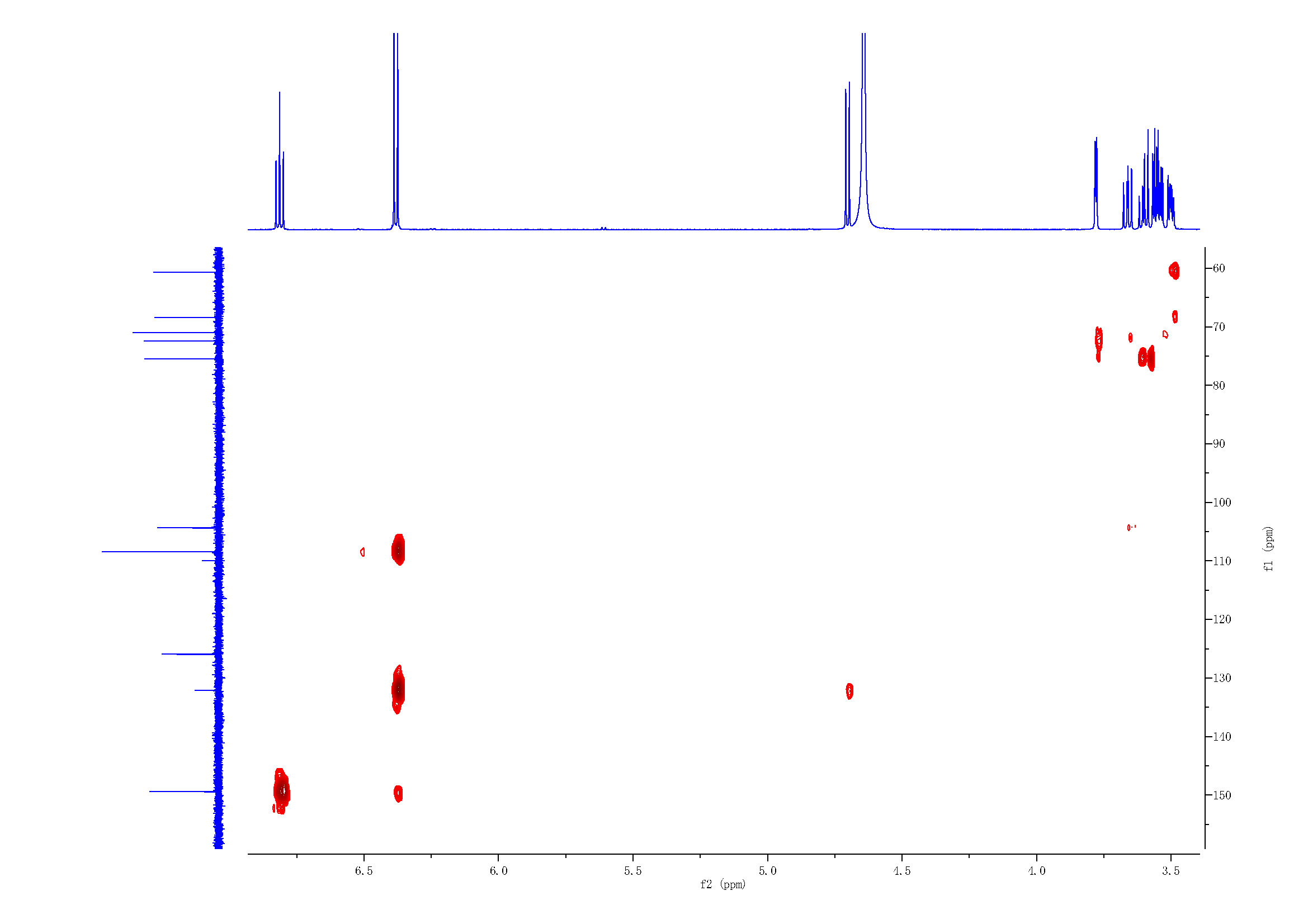

Supplement: S20 Fig — (TIF) [file pone.0121445.s020.tif]

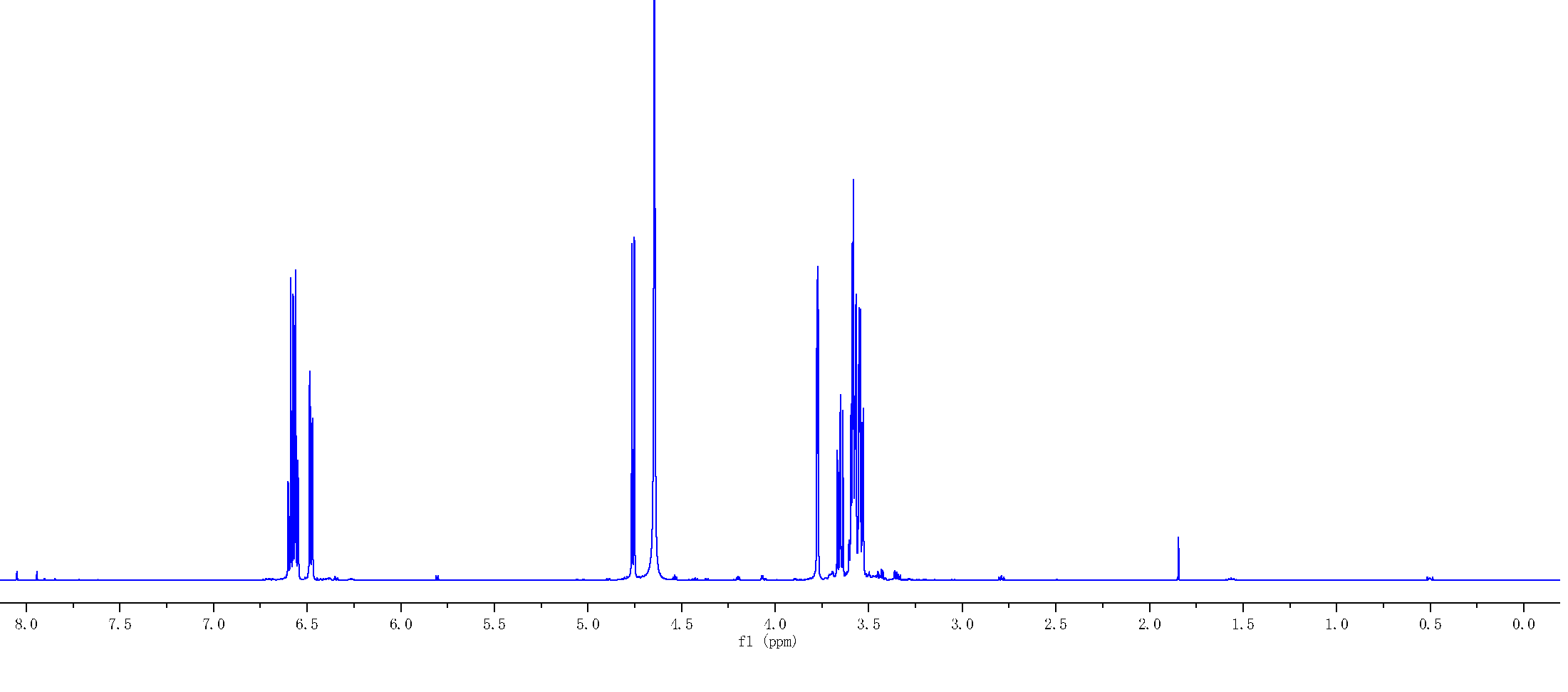

Supplement: S21 Fig — (TIF) [file pone.0121445.s021.tif]

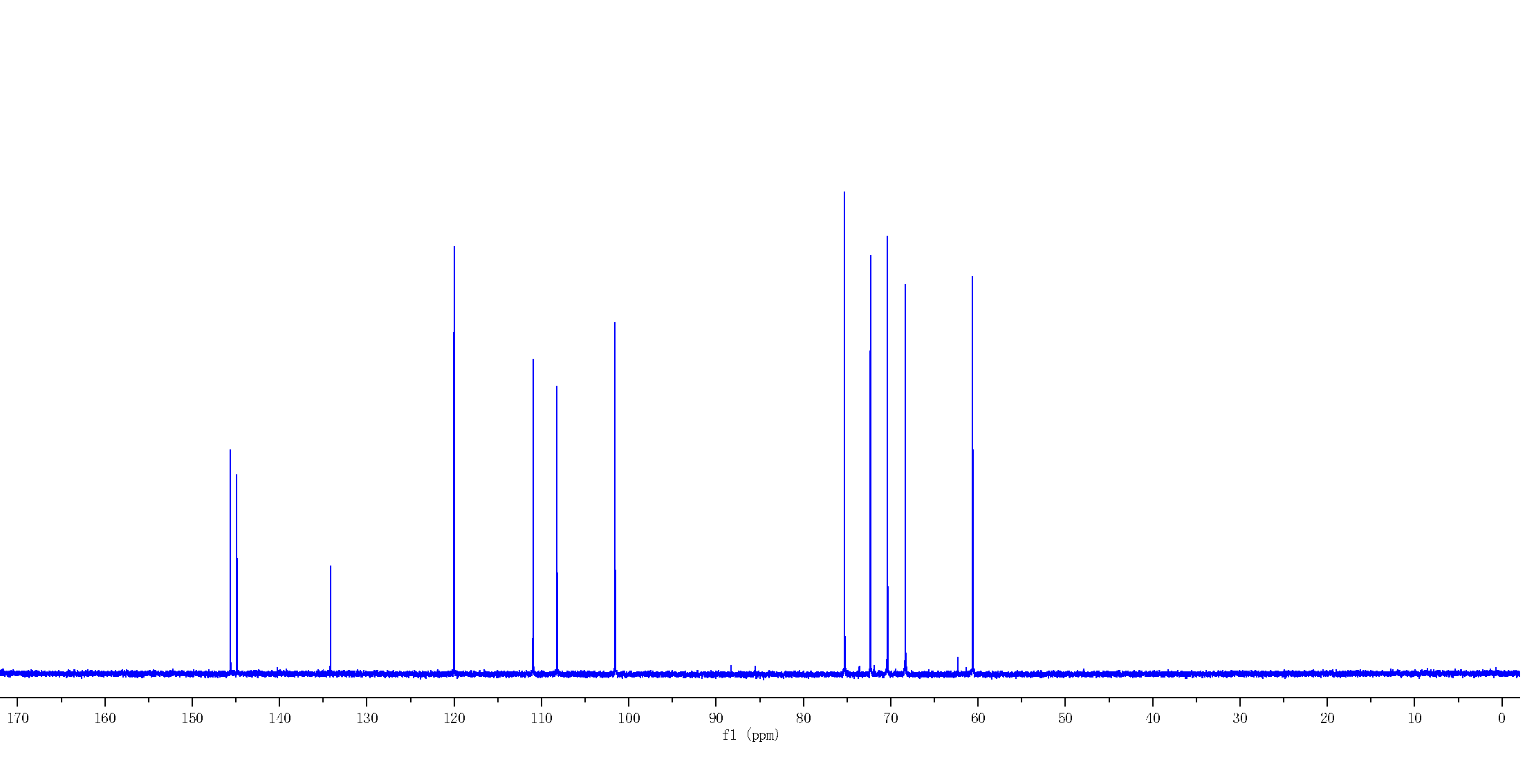

Supplement: S22 Fig — (TIF) [file pone.0121445.s022.tif]

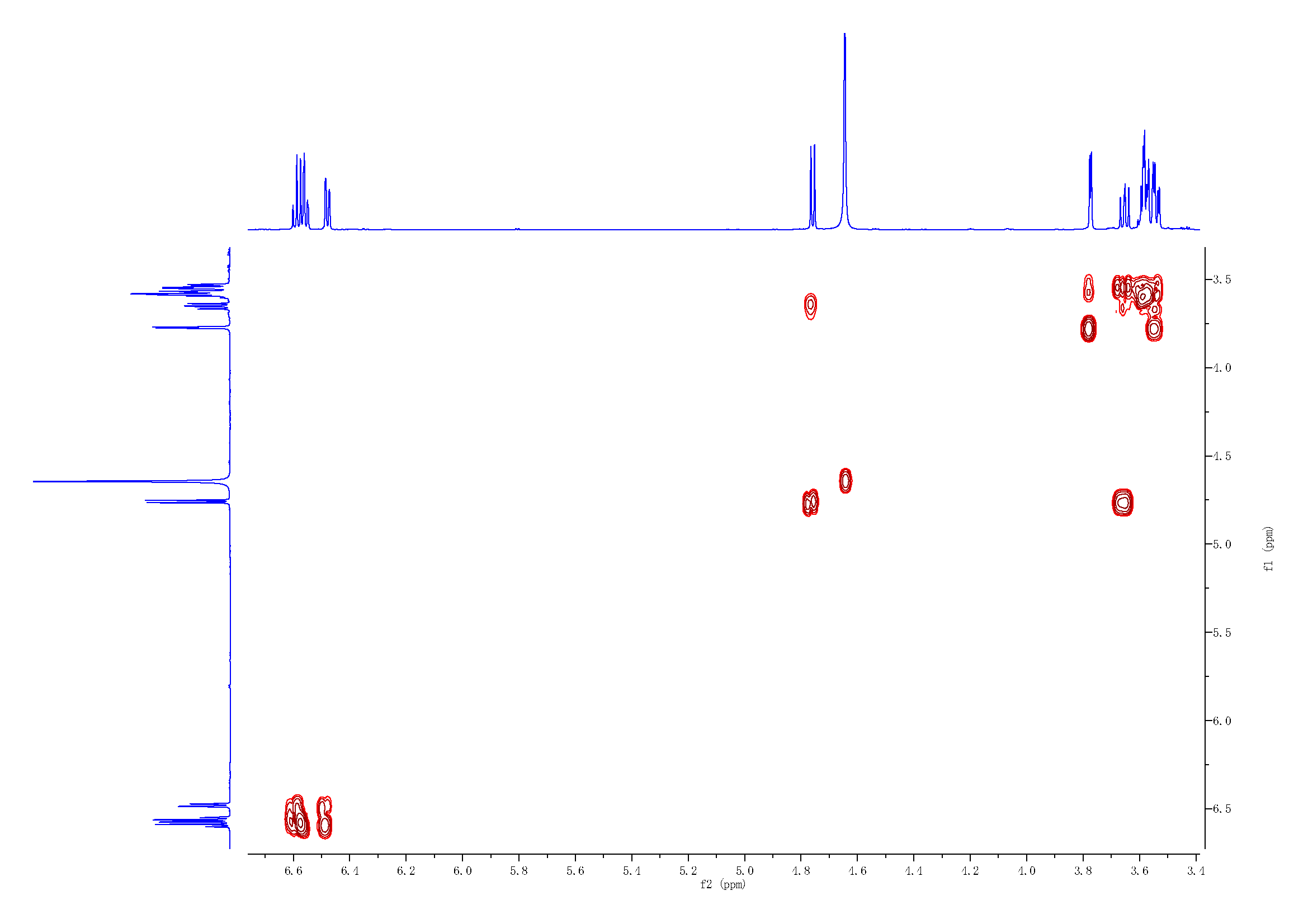

Supplement: S23 Fig — (TIF) [file pone.0121445.s023.tif]

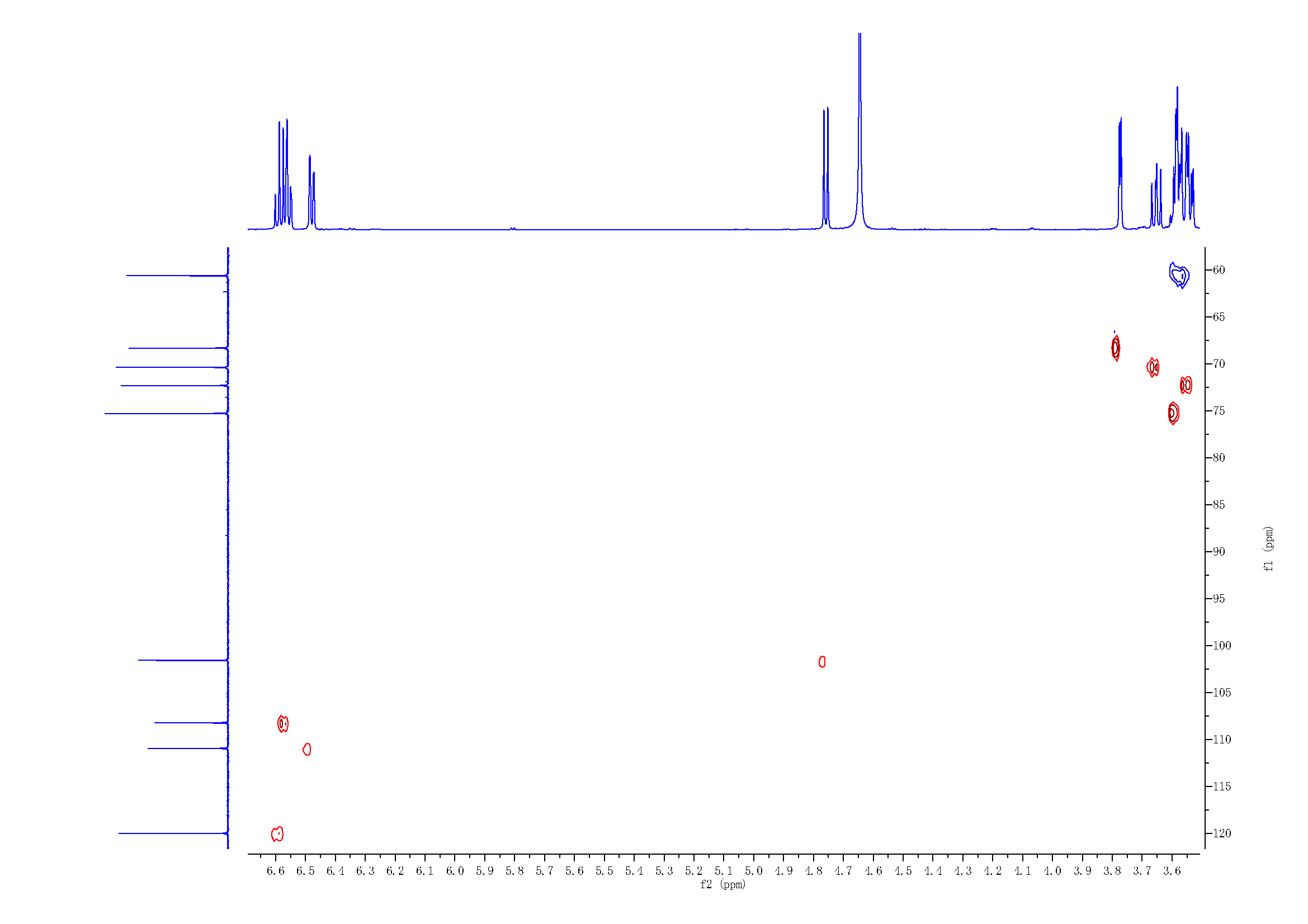

Supplement: S24 Fig — (TIF) [file pone.0121445.s024.tif]

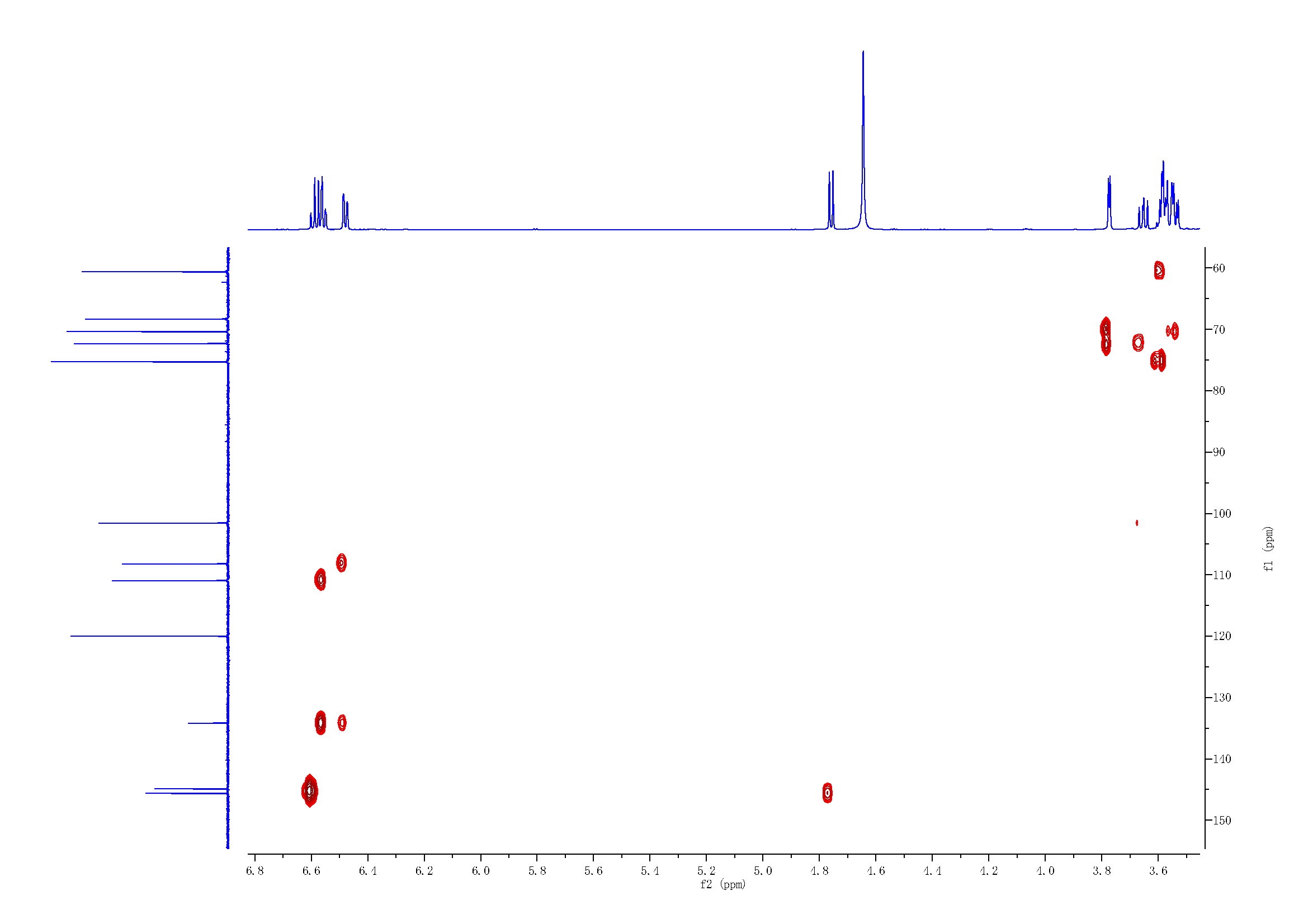

Supplement: S25 Fig — (TIF) [file pone.0121445.s025.tif]
