# Supplementary material for: Glycosylation of Phenolic Compounds by the Site-Mutated β-Galactosidase from Lactobacillus bulgaricus L3
Source: PLoS One. 2015 Mar 24;10(3):e0121445. doi: 10.1371/journal.pone.0121445 (PMC4372403; doi:10.1371/journal.pone.0121445)
Supplement: S1 Table — (DOC) [file pone.0121445.s026.doc]

Table S1 Primers used in site-directed mutagenesis

| Primers | Nucleotide sequences (5' to 3') |
| --- | --- |
| Ala-F  Ala-R | CGGGGATGACTCC**GCG**GGGCAGAAGGTCCA  **CGC**GGAGTCATCCCCGCCGACCCCCATCTG |
| Cys-F  Cys-R | CGGGGATGACTCC**TGC**GGGCAGAAGGTCCA  **GCA**GGAGTCATCCCCGCCGACCCCCATCTG |
| Asp-F  Asp-R | CGGGGATGACTCC**GAT**GGGCAGAAGGTCCA  **ATC**GGAGTCATCCCCGCCGACCCCCATCTG |
| Glu-F  Glu-R | CGGGGATGACTCC**GAA**GGGCAGAAGGTCCA  **TTC**GGAGTCATCCCCGCCGACCCCCATCTG |
| Phe-F  Phe-R | CGGGGATGACTCC**TTT**GGGCAGAAGGTCCA  **AAA**GGAGTCATCCCCGCCGACCCCCATCTG |
| Gly-F  Gly-R | CGGGGATGACTCC**GGC**GGGCAGAAGGTCCA  **GCC**GGAGTCATCCCCGCCGACCCCCATCTG |
| His-F  His-R | CGGGGATGACTCC**CAT**GGGCAGAAGGTCCA  **ATG**GGAGTCATCCCCGCCGACCCCCATCTG |
| Ile-F  Ile-R | CGGGGATGACTCC**ATT**GGGCAGAAGGTCCA  **AAT**GGAGTCATCCCCGCCGACCCCCATCTG |
| Lys-F  Lys-R | CGGGGATGACTCC**AAA**GGGCAGAAGGTCCA  **TTT**GGAGTCATCCCCGCCGACCCCCATCTG |
| Leu-F  Leu-R | CGGGGATGACTCC**CTG**GGGCAGAAGGTCCA  **CAG**GGAGTCATCCCCGCCGACCCCCATCTG |
| Met-F  Met-R | CGGGGATGACTCC**ATG**GGGCAGAAGGTCCA  **CAT**GGAGTCATCCCCGCCGACCCCCATCTG |
| Asn-F  Asn-R | CGGGGATGACTCC**AAC**GGGCAGAAGGTCCA  **GTT**GGAGTCATCCCCGCCGACCCCCATCTG |
| Pro-F  Pro-R | CGGGGATGACTCC**CCG**GGGCAGAAGGTCCA  **CGG**GGAGTCATCCCCGCCGACCCCCATCTG |
| Gln-F  Gln-R | CGGGGATGACTCC**CAG**GGGCAGAAGGTCCA  **CTG**GGAGTCATCCCCGCCGACCCCCATCTG |
| Arg-F  Arg-R | CGGGGATGACTCC**CGC**GGGCAGAAGGTCCA  **GCG**GGAGTCATCCCCGCCGACCCCCATCTG |
| Ser-F  Ser-R | CGGGGATGACTCC**AGC**GGGCAGAAGGTCCA  **GCT**GGAGTCATCCCCGCCGACCCCCATCTG |
| Thr-F  Thr-R | CGGGGATGACTCC**ACC**GGGCAGAAGGTCCA  **GGT**GGAGTCATCCCCGCCGACCCCCATCTG |
| Val-F  Val-R | CGGGGATGACTCC**GTG**GGGCAGAAGGTCCA  **CAC**GGAGTCATCCCCGCCGACCCCCATCTG |
| Tyr-F  Tyr-R | CGGGGATGACTCC**TAC**GGGCAGAAGGTCCA  **GTA**GGAGTCATCCCCGCCGACCCCCATCTG |
